# CDK4/6 inhibitors induce breast cancer senescence with enhanced anti‐tumor immunogenic properties compared with DNA‐damaging agents

**DOI:** 10.1002/1878-0261.13541

**Published:** 2023-11-02

**Authors:** Dong Hyun Lee, Muhammad Imran, Jae Ho Choi, Yoo Jung Park, Young Hwa Kim, Sunwoo Min, Tae Jun Park, Yong Won Choi

**Affiliations:** ^1^ Department of Biochemistry and Molecular Biology Ajou University School of Medicine Suwon Korea; ^2^ Department of Biomedical Sciences Ajou University Graduate School of Medicine Suwon Korea; ^3^ Inflamm‐Aging Translational Research Center Ajou University Medical Center Suwon Korea; ^4^ Department of Hematology‐Oncology Ajou University School of Medicine Suwon Korea; ^5^ Department of Biological Sciences Korea Advanced Institute of Science and Technology (KAIST) Daejeon Korea

**Keywords:** CDK4/6 inhibitors, DNA‐damaging agents, SASP, therapy‐induced senescence, tumor microenvironment

## Abstract

Since therapy‐induced senescence (TIS) can either support or inhibit cancer progression, identifying which types of chemotherapeutic agents can produce the strongest anti‐tumor TIS is an important issue. Here, cyclin‐dependent kinase4/6 inhibitors (CDK4/6i)‐induced senescence was compared to the TIS induced by conventional DNA‐damaging agents. Despite both types of agents eliciting a similar degree of senescence, we observed increased expression of the senescence‐associated secretory phenotype (SASP) and ligands related to pro‐tumor immunity (*IL6*, *CXCL8*, *TGFβ*, *CD274*, and *CEACAM1*) and angiogenesis (*VEGFA*) mainly in TIS induced by DNA‐damaging agents rather than by CDK4/6i. Additionally, although all agents increased the expression of anti‐tumor immunomodulatory proteins related to antigen presentation (*MHC‐I*, *B2M*) and T cell chemokines (*CXCL9*, *10*, *11*), CDK4/6i‐induced senescent cells still maintained this expression at a similar or even higher intensity than cells treated with DNA‐damaging agents, despite the absence of nuclear factor‐kappa‐B (NF‐κB) and p53 activation. These data suggest that in contrast with DNA‐damaging agents, which augment the pro‐tumorigenic microenvironment via pro‐inflammatory SASP, CDK4/6i can generate TIS only with antitumor immunomodulatory proteins.

## Graphical abstract

To identify which type of chemotherapeutic agent produces the strongest anti‐tumor therapy‐induced senescence (TIS), CDK4/6 inhibitors (CDK4/6i) were compared to conventional DNA‐damaging agents. Compared to DNA‐damaging agent‐induced senescent cancer cells, CDK4/6i‐induced senescent cancer cells clearly showed less prominent expression of the senescence‐associated secretory phenotype (SASP) and ligands associated with inflammation and angiogenesis, whereas they had comparable or even higher expression of ligands associated with anti‐tumor immunity.

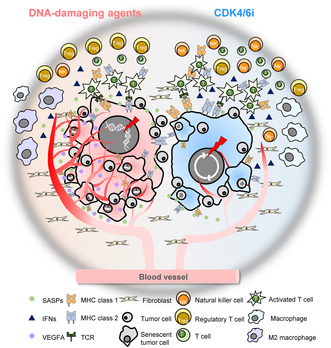

**Abbreviations**
AI: aromatase inhibitors
APCs: antigen‐presenting cells
BP: biological process
CC: cellular component
CDK: cyclin‐dependent kinase
CDK4/6i: cyclin‐dependent kinase inhibitors
Cis: cisplatin
DDR: DNA damage and repair
DEGs: differentially expressed genes
Doxo: doxorubicin
ELISA: enzyme‐linked immunosorbent assay
ET: endocrine therapy
GO: Gene ontology
GOBP: Gene ontology biological process
GOMF: Gene ontology molecular function
GSEA: Gene set enrichment analysis
IFN: interferons
IPA: ingenuity pathway analysis
ISG: interferon‐stimulated genes
Max: maximum
MDSC: myeloid‐derived suppressor cell
MEF: mouse embryonic fibroblast
MHC: major histocompatibility complex
Min: minimum
MMC: mitomycin
NES: normalized enrichment score
NF‐κB: nuclear factor‐kappa‐B
NK cells: natural killer cells
NKG2D: natural killer cells group 2 member D
Palbo: Palbociclib
PCR: polymerase chain reaction
PDAC: pancreatic ductal adenocarcinoma
PIDD: p53‐inducible death‐domain containing protein
RB: retinoblastoma protein
SAHF: senescence‐associated heterochromatin foci
SASP: senescence‐associated secretory phenotypes
SA‐β‐gal: senescence‐associated beta galactosidase
TGF‐β: transforming growth factor‐beta
TIS: therapy‐induced senescence
TME: tumor microenvironment
TNBC: triple‐negative breast cancer
TNFα: tumor necrosis factor‐alpha

## 1 Introduction

Therapy‐induced senescence (TIS), elicited by various chemotherapeutic agents and radiotherapy, has been regarded as one of the essential tumor suppressive mechanisms of current therapeutics [1, 2, 3]. However, the accumulation of senescent cells both in tumor and normal tissues can be detrimental by promoting tumor relapse, metastasis, and resistance to therapy by facilitating proliferation [4], angiogenesis [5], epithelial–mesenchymal transition [6], cell survival [7], and immune escape [8]. Moreover, senescent cells in normal tissue can contribute to therapy‐induced adverse effects [9]. The potential detrimental effects of TIS are mediated by the release of senescence‐associated inflammatory phenotype (SASP) that can modulate the tumor microenvironment (TME). Pro‐inflammatory SASP (IL‐1, IL‐6, CXCL8, TNFα) [10] and expression of pro‐angiogenic factors (VGEFA, GDF15) [11, 12] suppress anti‐tumor immunity, whereas some types of SASP can activate anti‐tumor immunity by augmenting tumor antigen presentation (Interferon family; IFN), cytotoxic T cell infiltration (CXCL9, CXCL10, CXCL11) [13], and activation of NK cells and T cells (IL‐2, MICB) [14, 15]. Moreover, senescent cells present in the TME can either facilitate or impede angiogenesis by releasing pro‐angiogenic (VEGFA) or anti‐angiogenic (MMRN2) factors [12, 16, 17], respectively. Therefore, it is of utmost importance to maximize the secretion of SASP linked to anti‐tumorigenic immunity and to minimize those that instigate pro‐tumorigenic immune responses. Hence, by better understanding the mechanisms that regulate pro‐ and anti‐tumorigenic SASP independently, it may be possible to develop more effective senescence inducers that preserve the ability to arrest the cell cycle and release SASP associated with the anti‐tumorigenic activity.

Since a prior study demonstrated that fibroblasts overexpressing p16^INK4A^ exhibit all the features of senescence but lack SASP [18], CDK4/6 inhibitors (CDK4/6i), as a mimic of p16^INK4A^ activation, can be a proper candidate of an ideal senescence inducer. However, unlike senescent fibroblasts overexpressing p16 ^INK4A^, CDK4/6i‐induced senescent cells excrete a range of SASP factors [19, 20]. Nevertheless, recent studies reveal that CDK4/6i treatment can elicit anti‐tumor immunity by targeting tumor cells and regulatory T cells and can also synergize with immune checkpoint inhibitors [21, 22, 23]. It is, therefore, still interesting to investigate whether senescent cancer cells induced by CDK4/6i treatment have a stronger ability to create the anti‐tumor immune microenvironment compared to other conventional chemotherapeutic DNA‐damaging agents. However, to the best of our knowledge, no systematic comparative study of the characteristics of DNA‐damaging agent‐induced and CDK4/6i‐induced senescent cancer cells, particularly in terms of the expression patterns of SASP and ligands that modulate the TME, has been conducted. Therefore, we examined the effects of representative DNA‐damaging agents (Cisplatin, Doxorubicin, Etoposide, and Carboplatin) and a CDK4/6i (Palbociclib, Abemaciclib) on the senescence of CDK4/6i‐sensitive breast cancer cells. We found that CDK4/6i‐induced senescent cancer cells induced strong expression of SASP and ligands related to an anti‐tumorigenic immunity, which was comparable with that induced by DNA‐damaging agent‐induced senescence. Furthermore, CDK4/6i led to significantly lower expression of SASP and ligands related to angiogenesis and pro‐tumorigenic immunomodulation.

## 2 Materials and methods

### 2.1 Cell culture and reagents

The breast cancer MCF‐7 (RRID: CVCL_0031, KCLB No. 30022, Korean Cell Line Bank, Seoul, Korea) and HCC1428 (RRID: CVCL_1253, KCLB No. 9S1428, Korean Cell Line Bank, Seoul, Korea) cells were cultured (1 × 10^5^ cells/60 mm culture dish) in RPMI 1640 (WELGENE, Gyeongsan, Korea) supplemented with 10% heat‐inactivated fetal bovine serum (FBS, GIBCO‐BRL, Grand Island, NY), 1% penicillin and streptomycin and maintained at 37 °C under 5% CO2 in a humidified atmosphere. The cell lines used in this study were authenticated by short‐tandem‐repeat profiling and were tested negative for mycoplasma contamination (е‐Myco™M plus Mycoplasma PCR Detection Kit, Cat. No 25237, Seongnam, Korea). DNA‐damaging agents including Doxorubicin, Cisplatin, Etoposide, and Carboplatin, CDK4/6i including Palbociclib, Abemaciclib, and Ribociclib, and aromatase inhibitors (AI) including Anastrozole, Letrozole (Selleck, Houston, TX) were treated to the cells. Antibodies purchased from Cell Signaling Technology (CST, Danvers, MA) include pRb (#9308), pAKT (#3787S), AKT (#9272), pErk1/2 (#9106S), Erk1/2 (#9107S), p‐p65 (#3031S), c‐Jun (#9165), p‐c‐Jun (#2361S), and p‐STAT3 (#4113S), Santa Cruz Biotechnology (SC, Dallas, TX); Rb (SC‐50), STAT3 (SC‐482), p53 (SC‐126), p21^Waf1/Cip1^ (SC‐6246), IκBα (SC‐1643), α‐Tubulin (SC‐32293), and β‐Actin (SC‐4778), Merck Millipore (Burlington, MA); anti‐γH2AX (05–636), Abcam (Cambridge, UK); anti‐Lamin B1 (ab16048).

### 2.2 Induction of cancer cell senescence

For the induction of senescence, MCF‐7 and HCC1428 cells were cultured and treated with DNA‐damaging agents (Cisplatin, Doxorubicin, Etoposide, and Carboplatin) and CDK4/6i (Palbociclib, Abemaciclib, and Ribociclib) for 2 days. The media were changed after every 2 days and cells were harvested following 5 days of exposure to DNA‐damaging agents and CDK4/6i.

### 2.3 Cell viability assay

MCF‐7 cells were exposed to various concentrations of Anastrozole and Letrozole (2.5, 5, 10, 20, and 30 μm). After 5 days of incubation with AI, cell viability assay was conducted using Cell Counting Kit‐8 (D‐Plus™ CCK cell viability assay kit, Cat. No. CCK‐3000, Donginbiotech Co., Ltd, Seoul, Korea) according to the manufacturer's instructions. Briefly, cells were treated with WST in fresh cultured medium and incubated for 1 h at 37 °C under 5% CO_2_ in a humidified atmosphere. Optical density was measured at 450 nm using an Eon™ microplate spectrophotometer (BioTEK, Winooski, VT). Cell viability was calculated in comparison with control MCF‐7.

### 2.4 SA‐β‐galactosidase staining

For SA‐β‐galactosidase staining, the control and drug‐treated MCF‐7 cells were washed twice with PBS, and then fixed with 4% paraformaldehyde for 5 min at room temperature in the dark. Cells were then washed three times with PBS and incubated with SA‐β‐gal staining solution (40 mm, pH 6 citrate/phosphate buffer, 5 mm each Ferro‐ and Ferricyanide, 150 mm NaCl, 2 mm MgCl_2_ and 1 mg·mL^−1^ X‐gal) for 15 h at 37 °C in the dark. After the incubation, cells were washed again with PBS and observed under a bright field microscope (OLYMPUS IX71, Tokyo, Japan).

### 2.5 Cell cycle analysis

The control and drug‐treated cells were harvested after 5 days of treatment, washed with PBS, and fixed in ice‐cold 70% (vol/vol) ethanol. Cells were treated with RNAase A and stained with propidium iodide. The distribution of the cell cycle was then analyzed using flow cytometry (BD FACSAria™ III, Franklin Lakes, NJ).

### 2.6 RNA extraction and qPCR

Total RNA was extracted from control and senescent MCF‐7 cells using the QIAGEN (Hilden, Germany) RNA extraction kit and reverse transcribed using a cDNA synthesis kit (Invitrogen, Waltham, MA). qPCR was performed using iQ SYBR® Green Supermix qPCR Kit (Bio‐Rad, Hercules, CA) on‐a CFX96™ Real‐Time System (Bio‐Rad, Hercules). Data were analyzed using the 2^(−ΔΔ*C*t)^ method. β‐actin was used as a control to normalize the expression of target genes. The primer sequences are given in Table S1.

### 2.7 Immunoblotting

The control and drug‐treated MCF‐7 cells were harvested after 5 days of treatment and lysed with RIPA buffer (50 mm Tris–HCl pH 7.5, 150 mm sodium chloride, 1 mm EDTA, 1% Nonidet P‐40, 1% sodium deoxycholic acid, 0.1% SDS along with protease and phosphatase inhibitors). Proteins were quantified and subjected to SDS/PAGE. Immunoblotting was performed on nitrocellulose membrane and proteins were detected using the immunoblotting detection kit WESTA SAVE^UP^™ (Seoul, Republic of Korea). The phosphorylated form and the total form of proteins on the same membrane were detected by stripping and reprobing the membrane. β‐actin and α‐tubulin were used as a loading control.

### 2.8 Enzyme‐linked immunosorbent assay (ELISA)

MCF‐7 cells (4 × 10^5^ cells/100 mm dish) were cultured and treated with DNA‐damaging agents and CDK4/6i for senescence induction, and the media were harvested after 5 days. Subsequently, the conditioned media were centrifuged and filtered through a 0.2‐μm pore size syringe filter. The secretions of VEGF, IL‐6, and CXCL8 were assessed in the conditioned media using a human DuoSet ELISA kit (R&D Systems, Minneapolis, MN) according to the manufacturer's instructions. The levels of VEGF, IL‐6, and CXCL8 in the conditioned media were quantified using Eon™ microplate spectrophotometer (BioTEK, Winooski, VT).

### 2.9 RNA sequencing and transcriptome analysis

RNA was isolated from control and senescent MCF‐7 cells using a QIAGEN extraction kit. RNA sequencing was carried out by Life & Science (LAS, Gimpo, South Korea). There were 60 million read‐pairs per sample, and gene mapping was performed using the altanalyze (version 2.0) alignment tool. Extracted fastq files were aligned with reference genome version hg38. The total RNA transcriptomes of 15 290 genes were normalized by the “EdgeR” package (version 3.40.2) in R studio. Before transcriptome analysis, read counts of 0 or < 10 in all 12 samples were considered pseudogenes and were filtered out. Trimmed mean of M values (TMM) was used as normalized values to analyze differentially expressed genes (DEGs).

### 2.10 Data processing and visualization

To illustrate gene expression patterns, we calculated *z*‐scores for 8832 DEGs. Sample correlations were evaluated using Spearman's rank correlation coefficient. To compare gene expression profiles across all samples, we visualized a similarity index heatmap based on *z*‐scores. These values were then input into Spearman's rank correlation analysis. For normalized enrichment score (NES) analysis, we utilized the R package “Fgsea” with 1000 permutations. Reference gene sets for various gene set enrichment analysis (GSEA) categories were obtained from Msigdbr in r. GSEA was performed both using the desktop application (GSEA 4.2.3) and TMM normalized values calculated through the “EdgeR” package. The following settings were used: collapse/Remap to gene symbols: collapse; permutation type: phenotype; chip platform: Human Ensemble Transcript ID MSigDB.vs.2023.1.Hs.Chip; enrichment statistic: weighted; metric for ranking genes: Signal2Noise; gene list sorting mode: real; gene list ordering mode: descending; maximum size of gene sets, 500; and minimum size of gene sets: 15. To compare overlapping signature gene sets between the control and drug‐induced senescence groups, cnetplots were generated using the “enrichplot” package and the “clusterprofiler” package. Ingenuity pathway analysis (IPA) was performed using qiagen IPA (version 42012434, Ingenuity Systems; Qiagen China Co., Ltd.) to identify pathways, top regulator effect networks, and gene signatures based on TMM values for drug‐treated MCF‐7 cells. All IPA analyses were conducted using a cutoff of *P* ≤ 0.05 and log 2‐fold change of ≥ 0.5.

### 2.11 Statistical analysis

All experiments were repeated at least three times and the data were presented as mean + SD. Statistical significance was calculated using one‐way ANOVA. *P* ≤ 0.05 was regarded as statistically significant. The Spearman and Pearson correlation coefficients were applied to assess the correlation between samples.

## 3 Results

### 3.1 A similar extent of breast cancer cell senescence is induced by DNA‐damaging agents and CDK4/6i

**Fig. 1:**
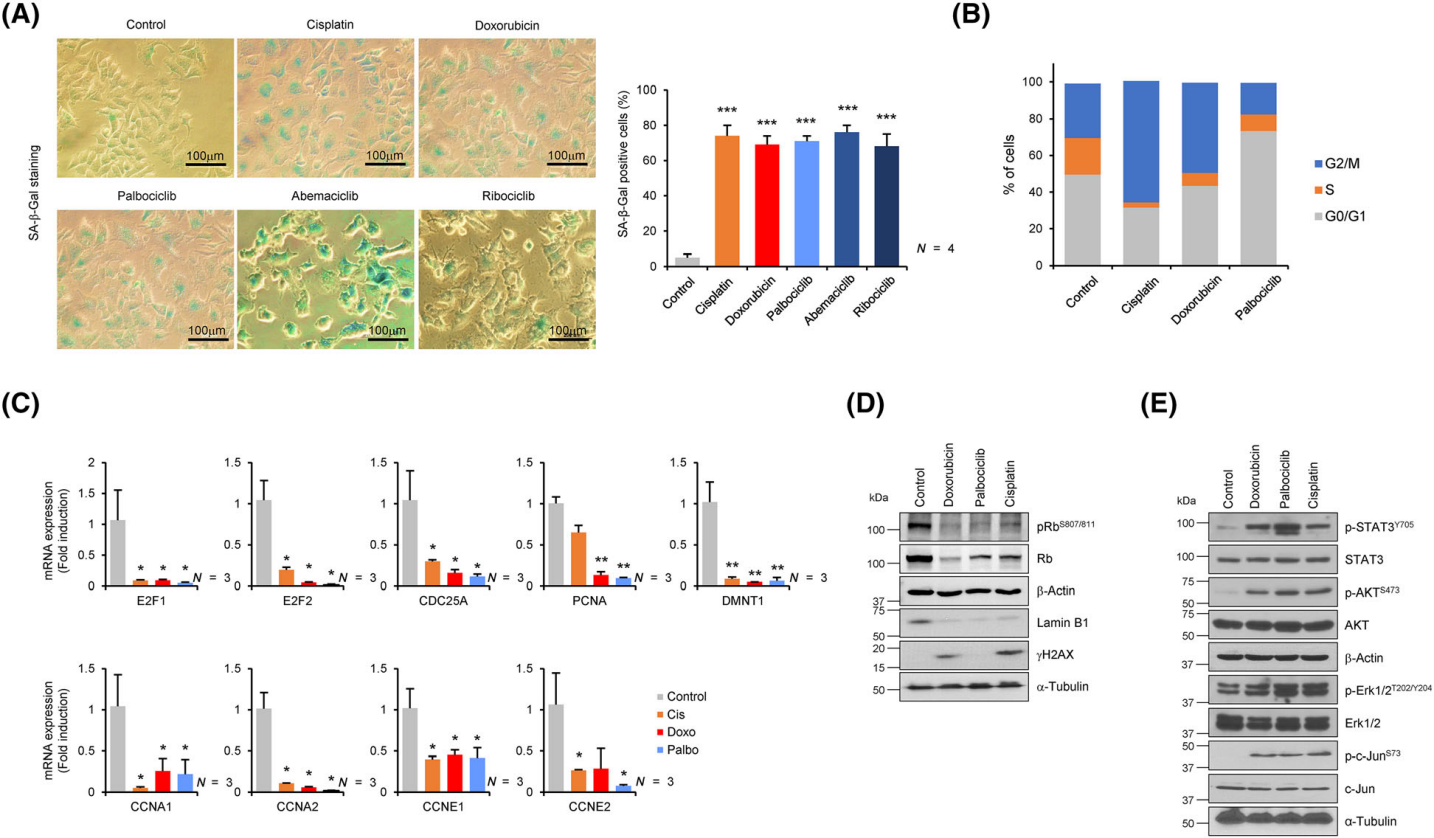
A similar extent of senescence induction by DNA‐damaging agents and CDK4/6i. (A) MCF‐7 cells were treated with DNA‐damaging agents (Cisplatin 5 μm and Doxorubicin 100 nm) and CDK4/6i (Palbociclib 2 μm, Abemaciclib 1 μm, and Ribociclib 5 μm) for 5 days and then SA‐β gal staining was performed. Representative images of SA‐β gal‐positive cells are shown. Scale bar, 100 μm. The percentage of stained cells was quantified and presented as a bar graph. Data are mean ± SD of four (*N* = 4) independent experiments. The *P*‐value was calculated using one‐way ANOVA. ***P < 0.001. (B) Cell cycle analysis of control, DNA‐damaging agents, and CDK4/6i‐treated senescent cells was performed by FACS. A representative bar graph shows the percentage of cells in each cell cycle phase. (C) The mRNA expression of genes was measured using RT‐qPCR in control, DNA‐damaging agents, and CDK4/6i‐induced senescent cells. The *P*‐value was calculated by the one‐way ANOVA. Data are mean ± SD of three (*N* = 3) independent experiments. *P < 0.05 and **P < 0.01. (D, E) The cells were harvested after 5 days of drug treatment and subjected to immunoblotting for protein expression analysis. β‐actin and α‐tubulin were used as loading controls. Data are representative of three (*N* = 3) independent experiments.

To characterize and compare senescence induced by treatment with DNA‐damaging agents (Cisplatin and Doxorubicin) and CDK4/6i, we screened various breast cancer cell lines to find out which could achieve stable senescent status for analysis post‐drug treatment. We selected MCF‐7 (hormone receptor (HR): positive/human epidermal growth factor receptor 2 (HER2): negative) breast cancer cells harboring wild‐type TP53 as a suitable model for studying the characteristics of TIS induced by different agents. In addition, since CDK4/6i are typically prescribed in conjunction with non‐steroidal AI for the treatment of HR‐positive and HER2‐negative breast cancer patients, we conducted an analysis to determine whether AI could influence the process of CDK4/6i‐induced senescence. Single treatments with AI at concentrations up to 30 μm demonstrated a slight but biologically insignificant compromise of cell viability (Fig. S1A), and combined treatment with AI and CDK4/6i revealed no additional effects on CDK4/6i‐induced senescence (Fig. S1B). Therefore, CDK4/6i were applied alone in subsequent experiments. Since various types of agents, and even various concentrations of the same agent, can induce a significantly different senescent status in the same cancer cell line, it is important to identify the optimal concentrations and treatment conditions for each drug to induce similar levels of TIS. After treatment with the indicated concentrations of drugs for the indicated times, we detected comparable levels of SA‐β‐gal activity induced by DNA‐damaging agents and CDK4/6i (Fig. 1A); however, the stage of cell cycle arrest was different; G2/M phase for DNA‐damaging agents and G0/G1 phase for CDK4/6i (Fig. 1B). All therapeutic agents reduced the expression of the E2F family and their well‐known target genes to a similar extent (Fig. 1C), along with phosphorylated retinoblastoma (RB) (Fig. 1D). In addition, reduced expression of Lamin B1, widely recognized as a biomarker of senescence, was observed to a similar degree after treatment with different agents, while increased phosphorylation of γH2AX was observed only in senescent cells induced by DNA‐damaging agents. This suggests that a meaningful DNA damage response was not activated in CDK4/6i‐induced senescent cancer cells (Fig. 1D). In addition, to investigate the possibility that DNA‐damaging agent‐ and CDK4/6i‐induced senescence activate distinct pathways, we analyzed other important signaling pathways activation. Regardless of TIS type, increased phosphorylation of JNK, Erk1/2, STAT3, and AKT suggested that the JNK, MAPK, JAK/STAT, and AKT pathways were commonly activated by TIS, although phosphorylation of STAT3 in CDK4/6i‐induced senescent cells was slightly stronger than in DNA‐damaging agent‐induced senescent cells (Fig. 1E). Overall, our data suggest that DNA‐damaging agents and CDK4/6i induce senescence in breast cancer cells to a similar extent.

### 3.2 Genes related to pro‐inflammatory response, anti‐tumor immunity, and angiogenesis are regulated differently in TIS by DNA‐damaging agents and CDK4/6i

**Fig. 2:**
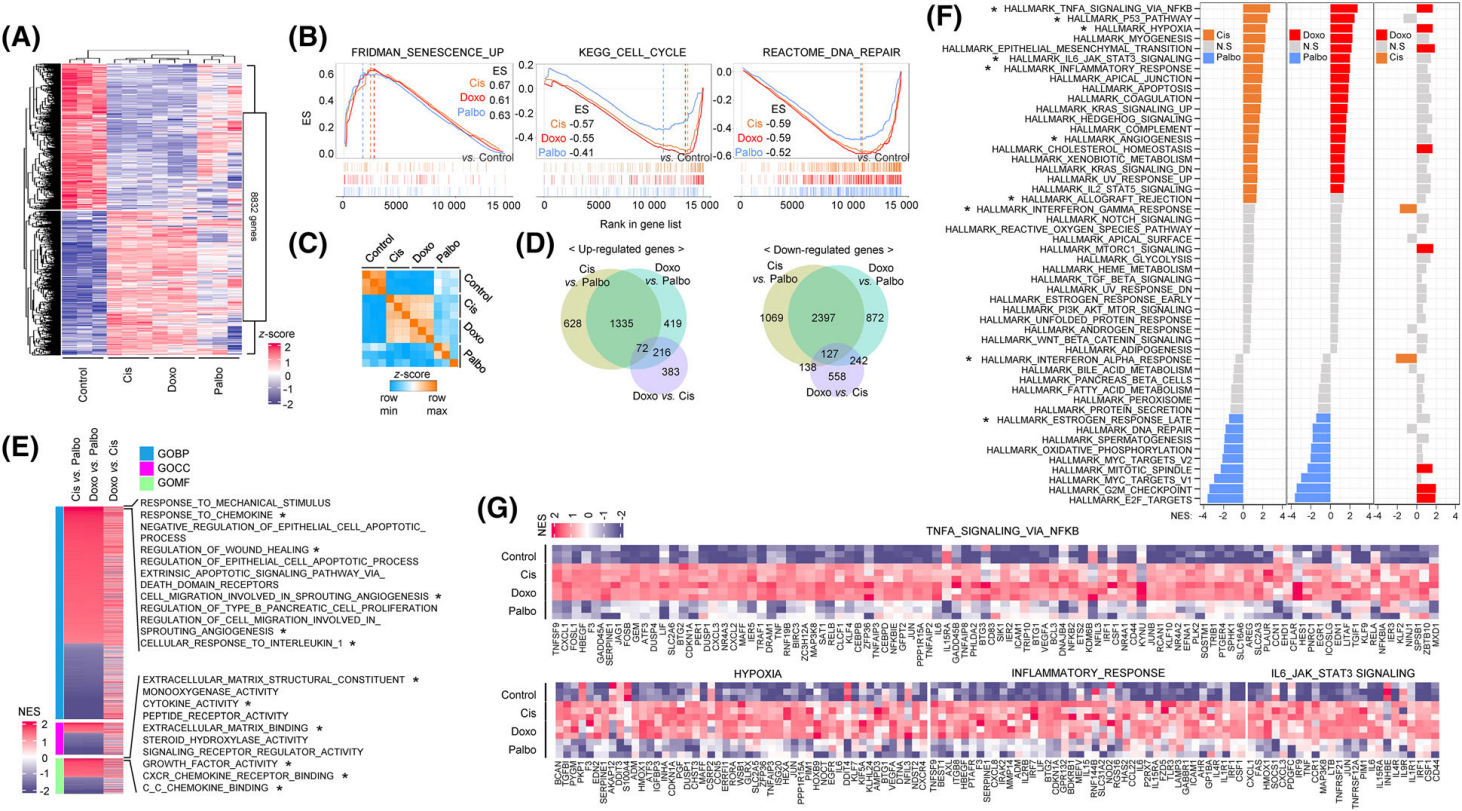
Differential expression of genes related to pro‐inflammatory response, anti‐tumor immunity, and angiogenesis induced by DNA‐damaging agents or CDK4/6i. (A) MCF‐7 cells were treated with DNA‐damaging agents (Cisplatin 5 μm and Doxorubicin 100 nm) and CDK4/6i (Palbociclib 2 μm) and RNA‐seq analysis was performed after 5 days. A heatmap was generated from the whole transcriptome of control and senescent cells, showing a hierarchical clustering correlation with a *z*‐score between the samples. Data are mean ± SD of three (*N* = 3) independent experiments. (B) Overlapping GSEA plots showing representative senescent gene sets including senescence, cell cycle, and DNA repair, with ES, in TIS compared to the control. (C) A similarity index matrix of the whole transcriptome comparing TIS and control. The color bar indicates the *z*‐score range, from the row min value to the row max value. (D) A Venn diagram showing significantly up‐ and downregulated genes between TIS groups. Light green: Cis vs. Palbo; sky blue: Doxo vs. Palbo; and purple: Doxo vs. Cis. (E) GO analysis of the whole transcriptome in TIS, presented as a heatmap. Significantly enriched top 10 gene sets indicated with an asterisk. (F) NES of HALLMARK gene sets in TIS are shown as a bar graph. Significantly enriched top 10 gene sets are indicated with an asterisk. (G) Heatmaps showing the leading edge of HALLMARK gene sets with *z*‐score in the whole transcriptome. Cis, Cisplatin; Doxo, Doxorubicin, Palbo, Palbociclib.

To clarify even slight differences in the expression of genes involved in not only SASP but also modulation of the TME, we examined changes in the whole transcriptome after TIS by using an RNA‐sequence alignment tool. The expression pattern of 8832 genes arranged by hierarchical clustering was displayed as a heatmap. Comparison of TIS samples with parental control cells revealed definitive changes in the transcriptome (Fig. 2A). The transcriptome analysis and GSEA of representative gene sets associated with general senescence showed that DNA‐damaging agents‐ and CDK4/6i‐induced senescence to a similar degree [24] (Fig. 2B). Despite the fact that all of the therapeutic agents altered expression of genes associated with TIS, we found differences in the expression patterns of genes induced by DNA‐damaging agents and CDK4/6i (Fig. 2A). Pearson's correlation similarity matrix analysis showed highly similar transcriptome patterns in senescent cells induced by DNA‐damaging agents, but global differences were observed between DNA‐damaging agent‐ and CDK4/6i‐induced senescence (Fig. 2C). Consistent with these results, the number of DEG between TIS by DNA‐damage agents and CDK4/6i were significantly higher than that of DEG between TIS by DNA damage agents (Fig. 2D). To identify cellular processes related to the, respectively, distinct patterns of gene expression in the senescence induced by different therapeutic agents, GSEA was performed with 33 591 gene sets (hallmark gene sets, curated gene sets, and ontology gene sets) as TIS vs. Control (Fig. S2). The gene ontology analysis with the comparison of TIS groups revealed that significantly distinctive cellular processes related to chemokines, angiogenesis, extracellular matrix structure, and cytokine activity were enriched in DNA‐damaging agent‐induced senescence (Fig. 2E). A 50 HALLMARK gene sets analysis revealed that gene sets potentially related to modulation of a pro‐tumorigenic microenvironment (e.g., TNF/NF‐κB signaling, hypoxia, inflammatory responses, and angiogenesis) was significantly enriched in TIS with DNA‐damaging agents compared to CDK4/6i. However, interestingly, NES of gene sets related to anti‐tumorigenic immunity (e.g., allograft rejection, interferon‐gamma responses, and interferon‐alpha responses) revealed no statistically significant difference between cells exposed to DNA‐damaging agents and CDK4/6i (Fig. 2F). Heatmaps of the leading edge in the HALLMARK gene sets; TNF/NF‐κB signaling, hypoxia, inflammatory responses, and IL‐6‐JAK‐STAT3 signaling, including well‐known pro‐inflammatory SASP (*CXCL1*, *CXCL2*, *CXCL3*, *CXCL8*, *CSF1*, *IL‐6*, *PLAUR*, *TNFα*, and *VEGFA*), clearly showed a significant difference in gene expression profiles between cells in which senescence was induced by DNA‐ damaging agents and CDK4/6i (Fig. 2G). These findings suggest that although the two different groups of therapeutic agents induce senescence to a similar extent, they regulate genes associated with inflammatory response and angiogenesis in different ways. Therefore, DNA‐damaging agent‐ and CDK4/6i‐induced senescence has the potential to modulate the TME differently. To support our hypothesis, we conducted a comprehensive analysis of three types of modulators of the TME: SASP and ligands related to pro‐ and anti‐tumorigenic immunity, and angiogenesis.

### 3.3 The pro‐tumorigenic secretome and associated ligands are expressed at higher levels by DNA‐damaging agent‐induced senescent cancer cells than CDK4/6i‐induced senescent cancer cells

**Fig. 3:**
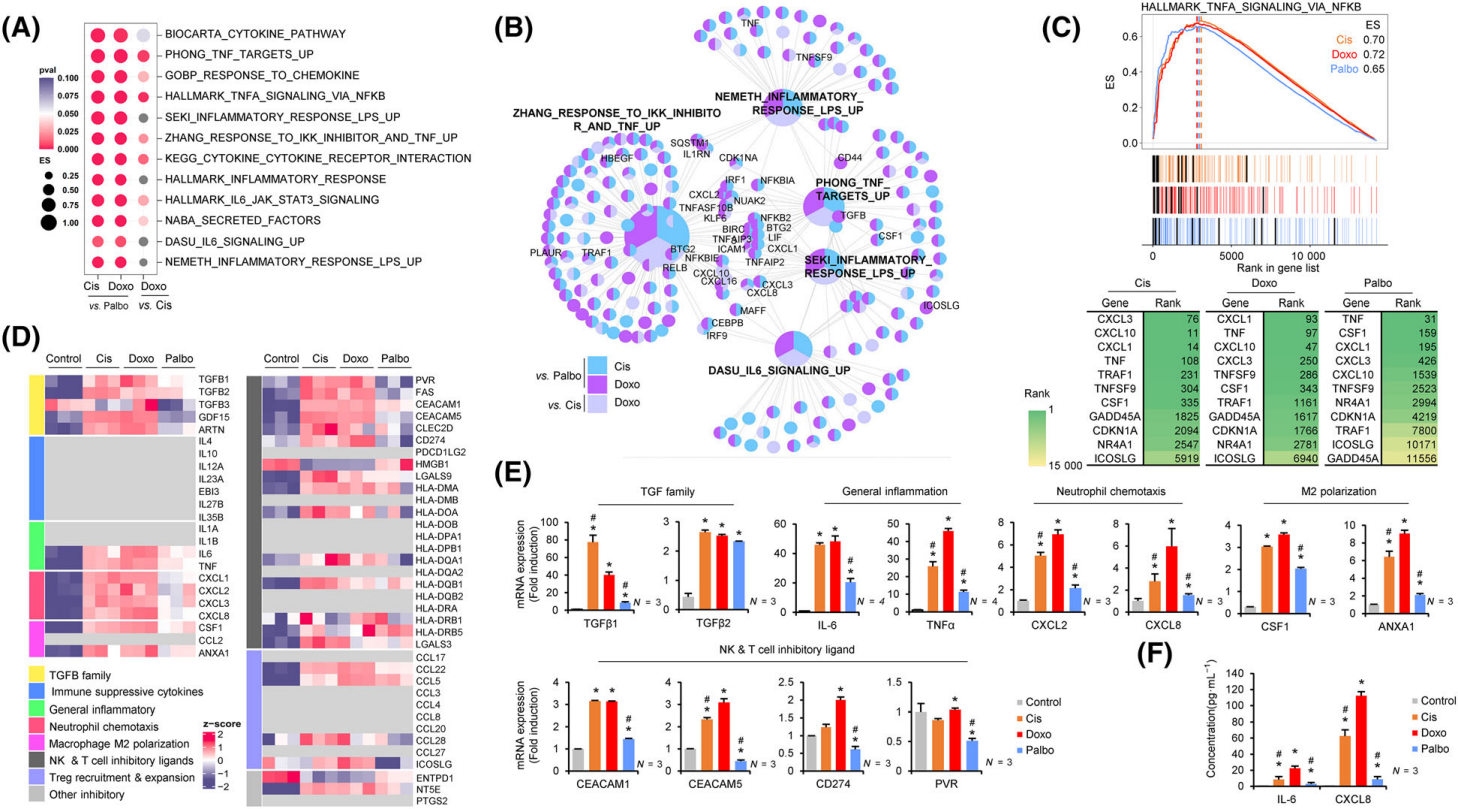
Differential expression of pro‐inflammatory SASP and ligands in DNA‐damaging agent‐induced senescent cancer cells compared to CDK4/6i‐induced senescent cancer cells. (A) A comparison of enrichment score (ES) of pro‐tumorigenic‐related gene sets among TIS groups presented as a dotplot with *P*‐value; Cis vs. Palbo, Doxo vs. Palbo, and Doxo vs. Cis. (B) A cnetplot of the gene sets selected from (A), showing significantly upregulated genes (log_2_FC ≥0.5, *P* ≤ 0.05) compared between the TIS groups; sky blue: Cis vs. Palbo; purple: Doxo vs. Palbo; and light purple: Doxo vs. Cis. The labeled genes on the cnetplot indicate pro‐tumorigenic secretome, ligands, and cytokines belonging to each gene set. (C) An overlapping GSEA presenting ES (upper panel), rank in gene list (middle panel) between TIS vs. control. Indicated genes as black bars in the middle panel arranged in tabulated form based on their rank. The green gradient indicates the ranks of the genes (bottom panel). (D) A heatmap of pro‐tumorigenic immune response‐related SASP expression, generated from the transcriptome and presented with *z*‐scores. The left panels of the heatmap indicate gene categories related to pro‐tumorigenic immune response, and the gray in the heatmap indicates unexpressed genes. (E) MCF‐7 cells were exposed to the DNA‐damaging agents and CDK4/6i and harvested after 5 days. The expression levels of mRNA encoding representative SASP, chemokines, and ligands related to pro‐tumorigenic immune responses were determined by RT‐qPCR. Data are mean ± SD of at least three (*N* ≥ 3) independent experiments. The *P*‐value was calculated using one‐way ANOVA. *P < 0.05 (TIS vs. Control) and ^#^ *P* < 0.05 (Cis vs. Doxo, Palbo vs. Doxo). (F) The cells (4 × 10^5^ cells/100 mm dish) were treated and harvested after 5 days. The concentrations (pg·mL^−1^) of IL‐6 and CXCL8 were measured by ELISA in conditioned media collected from senescent and control cells. Data are mean ± SD of three (*N* = 3) independent experiments. The *P*‐value was calculated using one‐way ANOVA. *P < 0.05 (TIS vs. Control) and ^#^ *P* < 0.05 (Cis vs. Doxo, Palbo vs. Doxo). Cis, Cisplatin; Doxo, Doxorubicin; Palbo, Palbociclib.

To investigate the possibility of differential expression of secretome and ligands associated with pro‐tumorigenic immune modulation by DNA‐damaging agent‐ and CDK4/6i‐induced senescent cancer cells, we performed GSEA using gene sets related to IL‐6, TNFα, cytokines, and inflammation and showed that enrichment score (ES) of gene sets was significantly higher in the DNA‐damaging agent‐ than in the CDK4/6i‐induced senescent cells (Fig. 3A and Fig. S2A). The cnetplot of DNA‐damaging agents vs. CDK4/6i showed that several pro‐inflammatory SASP, including *CXCL1*, *CXCL3*, *CXCL8*, *IL‐6*, *NF‐κB*, *TRAF1*, *TNFα*, and *TNFSF9*, were commonly upregulated among above gene sets (Fig. 3B). Although ES of the overlapping GSEA plot (HALLMARK_TNF_SIGNALING_VIA_NF‐ΚB) in DNA‐damaging agent‐induced senescence was slightly higher than in CDK4/6i‐induced senescence, the rank of representative pro‐inflammatory and tumorigenic immune response genes was significantly higher in DNA‐damaging agent‐induced senescent cells (Fig. 3C). Consistent with the results from the above GSEA, the transcriptome data showed that expression of most secretory proteins and ligands associated with pro‐tumorigenic immune modulation which were categorized based on literature was upregulated in DNA‐damaging agent‐induced senescent cells than in CDK4/6i‐induced senescent cells (Fig. 3D). Real‐time qPCR further confirmed that transcripts of genes involved in the pro‐tumorigenic immune response, such as TGFβ family (*TGFβ1* and *TGFβ2*), inflammatory cytokines (*IL‐6*, *TNFα*), neutrophil chemokines (*CXCL2*, *CXCL8*), macrophage M2 polarization inducers (*CSF1* and *ANXA1*), and NK and T‐cell inhibitory ligands (*CEACAM1*, *CEACAM5*, *PVR*, and *CD274*), were more abundantly expressed in DNA‐damaging agent‐induced senescent cells (Fig. 3E). Protein levels of IL‐6 and CXCL8 in conditioned medium from DNA‐damaging agent‐induced senescent cells were also significantly higher than in that from CDK4/6i‐induced senescent cells (Fig. 3F). Taken together, these data indicate that DNA‐damaging agent‐induced senescence in breast cancer cells leads to more robust expression of various pro‐inflammatory SASP (*TGFβ1*, *TGFβ2*, and *IL‐6*, *CXCL2*, *CXCL8*, *CSF1*) and cell surface ligands (*CEACAM1*, *CEACAM5*, *PVR*, and *CD274*) that augment pro‐tumorigenic immune responses within the TME.

### 3.4 Pro‐angiogenic factors are more abundantly expressed by DNA‐damaging agent‐induced senescent cancer cells than by CDK4/6i‐induced senescent cancer cells

**Fig. 4:**
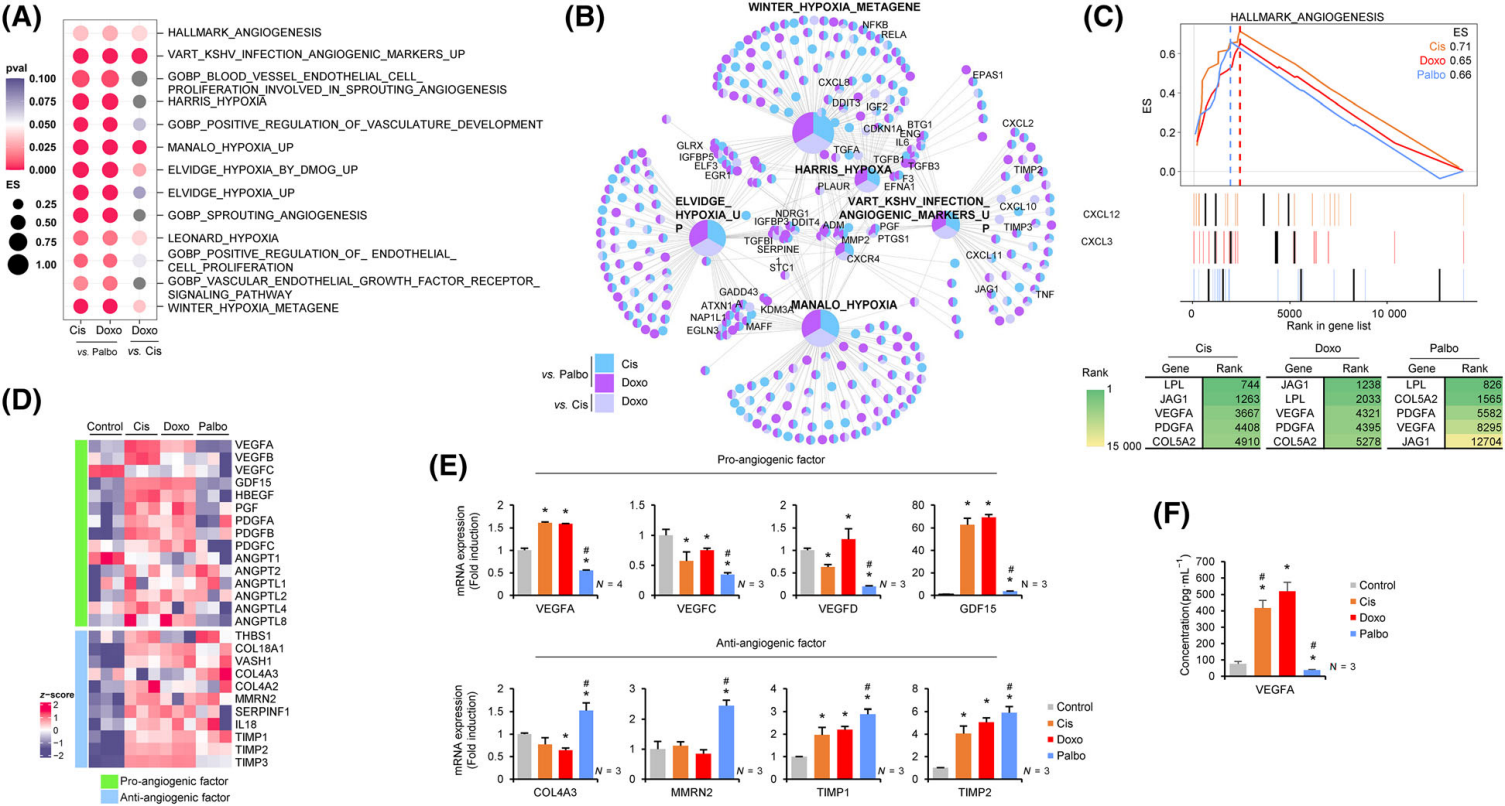
Pro‐angiogenic and anti‐angiogenic factors are enriched in DNA‐damaging agent‐ and CDK4/6i‐induced senescent cells, respectively. (A) A comparison of enrichment score (ES) of pro‐angiogenic gene sets among TIS groups presented as a dotplot with *P*‐value; Cis vs. Palbo, Doxo vs. Palbo, and Doxo vs. Cis. (B) A cnetplot of the gene sets selected from (A), showing a comparison of the significantly upregulated genes (log_2_FC ≥0.5, *P* ≤ 0.05) between TIS groups; sky blue: Cis vs. Palbo; purple: Doxo vs. Palbo; and light purple: Doxo vs. Cis. The labeled genes on the cnetplot indicate angiogenic factors belonging to each gene set. (C) An overlapping GSEA showing ES (upper panel) and rank in gene list (middle panel) between TIS vs. Control. Indicated genes are shown as black bars (middle panel) arranged in tabulated form based on rank. The green gradient indicates the ranks of the genes (bottom panel). (D) A heatmap of angiogenic factors was obtained from the transcriptome and presented as *z*‐scores. The left panels of the heatmap indicate categories of pro‐ and anti‐angiogenic factors, and the gray in the heatmap indicates unexpressed genes. (E) MCF‐7 cells were treated with the agents and harvested after 5 days. The expression levels of pro‐angiogenic and anti‐angiogenic genes were determined by RT‐qPCR. Data are mean ± SD of at least three (*N* = 3) independent experiments. The *P*‐value was calculated using one‐way ANOVA. *P < 0.05 (TIS vs. Control) and ^#^ *P* < 0.05 (Cis vs. Doxo, Palbo vs. Doxo). (F) The cells (4 × 10^5^ cells/100 mm dish) were treated and harvested after 5 days. The concentration (pg·mL^−1^) of VEGF was measured by ELISA in conditioned media collected from senescent and control cells. Data are mean ± SD of three (*N* = 3) independent experiments. The *P*‐value was calculated using one‐way ANOVA. *P < 0.05 (TIS vs. Control) and ^#^ *P* < 0.05 (Cis vs. Doxo, Palbo vs. Doxo). Cis, Cisplatin; Doxo, Doxorubicin; Palbo, Palbociclib.

Based on the finding that DNA‐damaging agent‐induced senescent cancer cells express high levels of VEGF (Fig. 2G), we investigated whether proteins secreted in the DNA‐damaging agent‐induced senescence are associated with angiogenesis. GSEA showed that gene sets related to angiogenesis, vascular endothelial cells, and hypoxia were significantly enriched in senescent cells induced by DNA‐damaging agents (Fig. 4A and Fig. S2B). Among these gene sets, the commonly regulated genes were analyzed by cnetplot (Fig. 4B), revealing that the expression ranks of these genes were significantly lower in CDK4/6i‐induced senescence (Fig. 4C). The heatmap of RNA‐seq data for pro and anti‐angiogenic factors revealed that levels of pro‐angiogenic factors such as *VEGFA* [16], *HBEGF* [25, 26], *PGF* [27], the *PDGF* family [28, 29, 30, 31], and the *ANGPTL* family [32, 33, 34, 35, 36] were higher in DNA‐damaging agent‐induced senescent MCF‐7 cells, while some anti‐angiogenic factors were significantly upregulated in CDK4/6i‐induced senescent cells (Fig. 4D). Real‐time qPCR analysis demonstrated that expression of pro‐angiogenic factors such as *VEGFA* and *GDF‐15* [11, 12, 16] increased significantly in DNA‐damaging agent‐induced senescent cells, whereas CDK4/6i‐induced senescent cells showed significant upregulation of anti‐angiogenic factors such as *COL4A3* [37, 38], *MMRN2* [17], *TIMP1* [39], and *TIMP2* [40], along with a consistent trend toward the decreased expression of VEGFA (Fig. 4E). To confirm the reciprocal expression pattern of *VEGFA* mRNA as a master regulator of angiogenesis between DNA‐damaging agent‐ and CDK4/6i‐induced senescent cells, we measured the levels of VEGFA protein in a conditioned medium by ELISA. Consistent with mRNA expression data, the secretion level of VEGFA in conditioned medium from CDK4/6i‐induced senescent cells was significantly lower than those in conditioned medium from non‐senescent cells (Fig. 4F). Taken together, these findings suggest that DNA‐damaging agent‐induced senescence can promote tumor angiogenesis via secretion of pro‐angiogenic factors, while CDK4/6i‐induced senescence can inhibit angiogenesis through regulation of anti‐angiogenic factors in the TME.

### 3.5 The antigen presentation and interferon signaling activities of CDK4/6i‐induced senescent cells are comparable with those of DNA‐damaging agent‐induced senescent cells

**Fig. 5:**
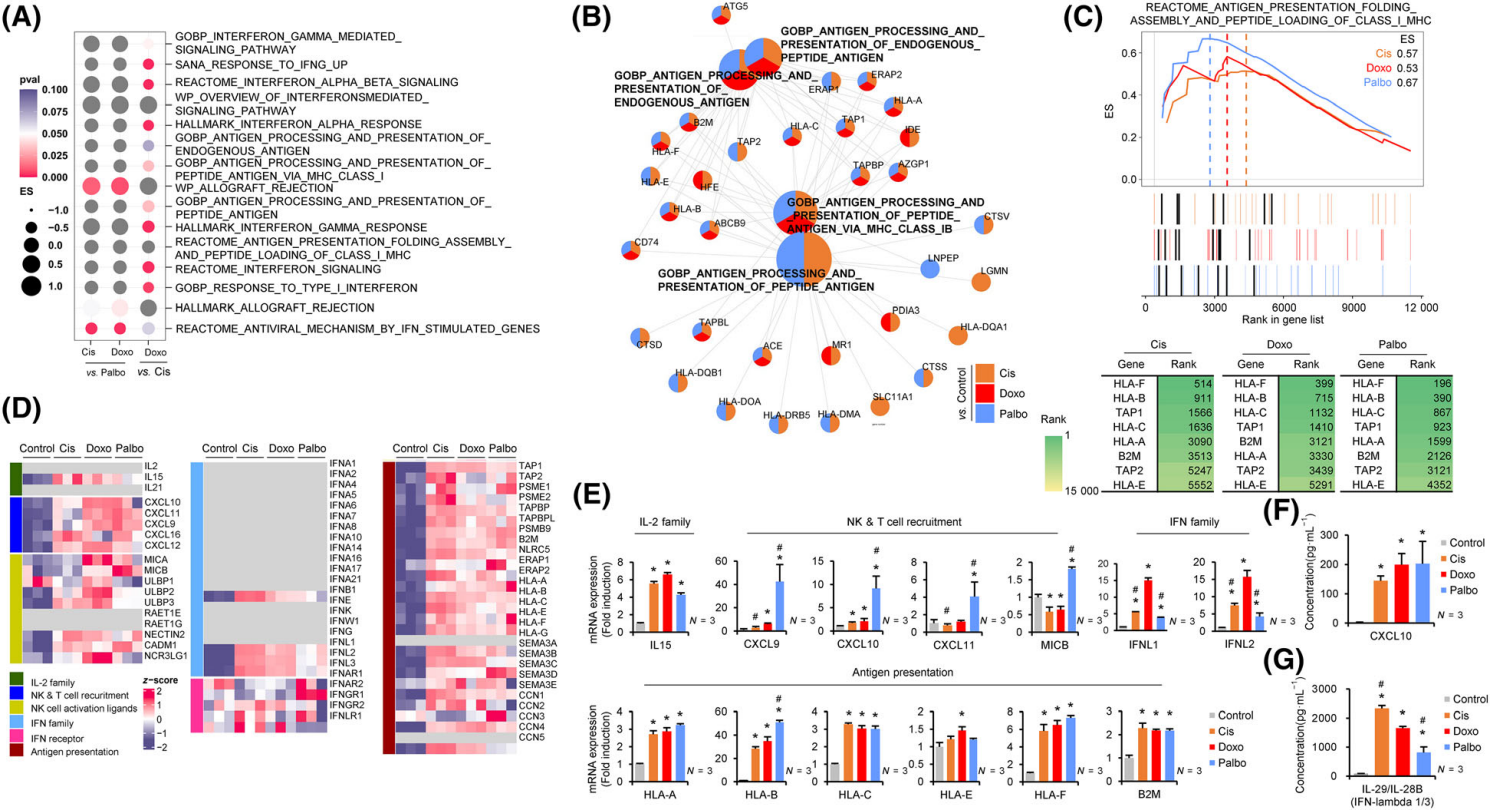
A similar antigen presentation and interferon signaling activities in DNA‐damaging agent‐ and CDK4/6i‐induced senescent cells. (A) A comparison of ES of anti‐tumorigenic immune response‐related gene sets among TIS groups with *P*‐value; Cis vs. Palbo, Doxo vs. Palbo, and Doxo vs. Cis. (B) A cnetplot of the gene sets selected from (A), showing a comparison of the significantly upregulated genes (log_2_FC ≥0.5, *P* ≤ 0.05) of TIS groups vs. Control; orange: Cis vs. Control; red: Doxo vs. Control; and blue: Doxo vs. Control. The labeled genes in the cnetplot indicate genes associated with antigen presentation within each gene set. (C) An overlapping GSEA showing ES (upper panel), rank in gene list (middle panel) between TIS vs. Control. Indicated genes as black bars (middle panel) arranged in tabulated form based on their rank. The green gradient indicates the ranks of the gens (bottom panel). (D) A heatmap of anti‐tumorigenic immune response factors was obtained from the transcriptome and presented as *z*‐scores. The left panels of the heatmap indicate the categories of anti‐tumorigenic immune response factors, and the gray in the heatmap indicates unexpressed genes. (E) MCF‐7 cells were treated with the agents and harvested after 5 days. The expression levels of anti‐tumorigenic were determined by RT‐qPCR. Data are mean ± SD of three (*N* = 3) independent experiments. The *P*‐value was calculated by the one‐way ANOVA. *P < 0.05 (TIS vs. Control) and ^#^ *P* < 0.05 (Cis vs. Doxo, Palbo vs. Doxo). (F, G) The cells (4 × 10^5^ cells/100 mm dish) were treated and harvested after 5 days. The concentrations (pg·mL^−1^) of CXCL10 and IFN‐λ were measured by ELISA in conditioned media collected from senescent and control cells. Data are mean ± SD of three (*N* = 3) independent experiments. The *P*‐value was calculated using one‐way ANOVA. *P < 0.05 (TIS vs. Control) and ^#^ *P* < 0.05 (Cis vs. Doxo, Palbo vs. Doxo). Cis, Cisplatin; Doxo, Doxorubicin; Palbo, Palbociclib.

As shown in Fig. 2F, we did not find a significant difference in gene sets related to T‐cell‐mediated anti‐tumor immunity between the TIS groups. This indicates that the capacity of CDK4/6i‐induced senescence to activate anti‐tumor immunity is probably similar to that of DNA‐damaging agent‐induced senescence, despite the reduced secretion of pro‐inflammatory and pro‐angiogenic SASP in the CDK4/6i‐group. GSEA analysis of gene sets related to allograft rejection, T‐cell‐mediated cytotoxicity, and antigen presentation revealed no statistical differences between the therapeutic agents, supporting the above observations (Fig. 5A and Fig. S2C). The cnetplot with above gene sets revealed that senescent cancer cells induced by all three agents increased expression of antigen presentation factors (Fig. 5B). Moreover, the overlapping GSEA showed that the rank of genes related to antigen presentation (*HLA‐F*, *HLA‐B*, *HLA‐C*, *TAP1*, and *B2M*) [41, 42, 43, 44, 45, 46, 47] in the CDK4/6i‐group was slightly higher compared to that in the DNA‐damaging agent group (Fig. 5C). The heatmap for genes involved in activating anti‐tumor immune responses showed increased expression of T‐cell chemokines (*CXCL9*, *CXCL10*, and *CXCL11*) [48, 49, 50, 51, 52], antigen processing proteins (*TAP1*, *TAP2*, *TAPBP*, *ERAP1*, and *ERAP2*) [44, 45, 53, 54, 55, 56], and MHC class I molecules (*HLA*s and *B2M*) in both DNA‐damaging agent‐ and CDK4/6i‐induced senescent cells, indicating activation of anti‐tumor immunity in both the groups (Fig. 5D). These results are consistent with those of recent studies [57, 58]. To validate the RNA‐seq results for anti‐tumor immunity, we performed RT‐qPCR on independent experimental samples to detect representative genes (Fig. 5E). Consistent with RNA‐seq data, all three types of senescent cancer cells showed increased expression of genes related to anti‐tumor immunity. Interestingly, increased expression of *CXCL9*, *CXCL10*, *CXCL11*, *MICB*, *HLA‐B*, and *HLA‐F* was more pronounced in CDK4/6i‐induced senescent cells than in DNA‐damaging agent‐induced senescent cells. Consistent with the mRNA data, the CXCL10 protein level in the conditioned medium derived from CDK4/6i‐induced senescence exhibited comparable or slightly elevated levels in comparison with DNA‐damaging agent‐induced senescence. IFN signaling activates downstream target genes involved in antigen presentation [49, 59, 60] as well as expression of T‐cell chemokines [61, 62]. Previous studies demonstrate that intracellular antiviral defense mechanisms could be triggered during cellular senescence through endogenous retroviral activation, resulting in *IFN* expression [63, 64]. Transcriptome analysis revealed no marked expression of basal mRNA transcripts of *IFN*‐α and *IFN*‐γ in MCF‐7 cells, with no increase observed after TIS. However, there was a significant increase in mRNA transcripts of *IFN*‐β (*IFNB1*) and *IFN‐*λ (*IFNL1*, *IFNL2*, and *IFNL3*) by TIS. Furthermore, the secreted protein level of *IFN‐*λ in conditioned medium from senescent cells was significantly higher than that in conditioned medium from non‐senescent cells (Fig. 5F). These findings suggest that TIS triggers the expression of genes related to anti‐tumor immune activation. CDK4/6i‐induced senescence also has this ability, at levels comparable to or even greater than that of DNA‐damaging agents.

For further confirmation of the above observations from MCF‐7 cells, another breast cancer cell line, HCC1428 (HR: positive/HER2: negative) [65], was used for TIS by DNA‐damaging agents and CDK4/6i. Similar to MCF‐7, SA‐β‐gal activity (Fig. S3A) and reduced expression of E2F target genes (Fig. S3B) showed that a comparable extent of senescence was induced by different types of agents. Increased phosphorylation of γH2AX was also observed only in senescent cells induced by DNA‐damaging agents (Fig. S3C). Increased phosphorylation of c‐Jun, Erk1/2, STAT3, and AKT after TIS and slightly stronger phosphorylation of STAT3 in CDK4/6i‐induced senescent cells compared to those of DNA‐damaging agent‐induced senescent cells was also observed in HCC1428 cells (Fig. S3D). In HCC1428 cells, DNA‐damaging agent‐induced senescence showed higher mRNA (Fig. S3E,G) and protein levels (Fig. S3H) of pro‐inflammatory SASP (*TGFβ1*, *IL‐6*, *CXCL8*, and *ANXA1*) and pro‐angiogenic factors (*VEGFA*, *VEGFB*, and *GDF15*) compared to those of CDK4/6i‐induced senescence. While consistent with data from MCF‐7, the expression level of genes related to anti‐tumor immunity such as T‐cell chemokines (*CXCL9*, *CXCL10*, and *CXCL11*) and antigen presentation (*HLA*s and *B2M*) in CDK4/6i‐induced senescent cells is similar or even higher than those of DNA‐damaging agent‐induced senescent cells (Fig. S3G), consistent with the mRNA data, in HCC1428, CXCL10 and *IFN‐*λ protein level in the conditioned medium derived from CDK4/6i‐induced senescence was also higher than those of DNA‐damaging agent‐induced senescence (Fig. S3H). In addition, senescent cancer cells by other types of DNA damaging agents (Etoposide and Carboplatin) (Fig. S4A,B) and CDK4/6i (Abemaciclib) also showed similar trends of mRNA expression pattern in pro‐inflammatory SASP (Fig. S4C), pro‐angiogenic factors (Fig. S4D), and anti‐tumor immune‐related genes (Fig. S4F).

### 3.6 DNA‐damaging agent‐induced senescence activates p53 and NF‐κB signaling significantly more strongly than CDK4/6i‐induced senescence

**Fig. 6:**
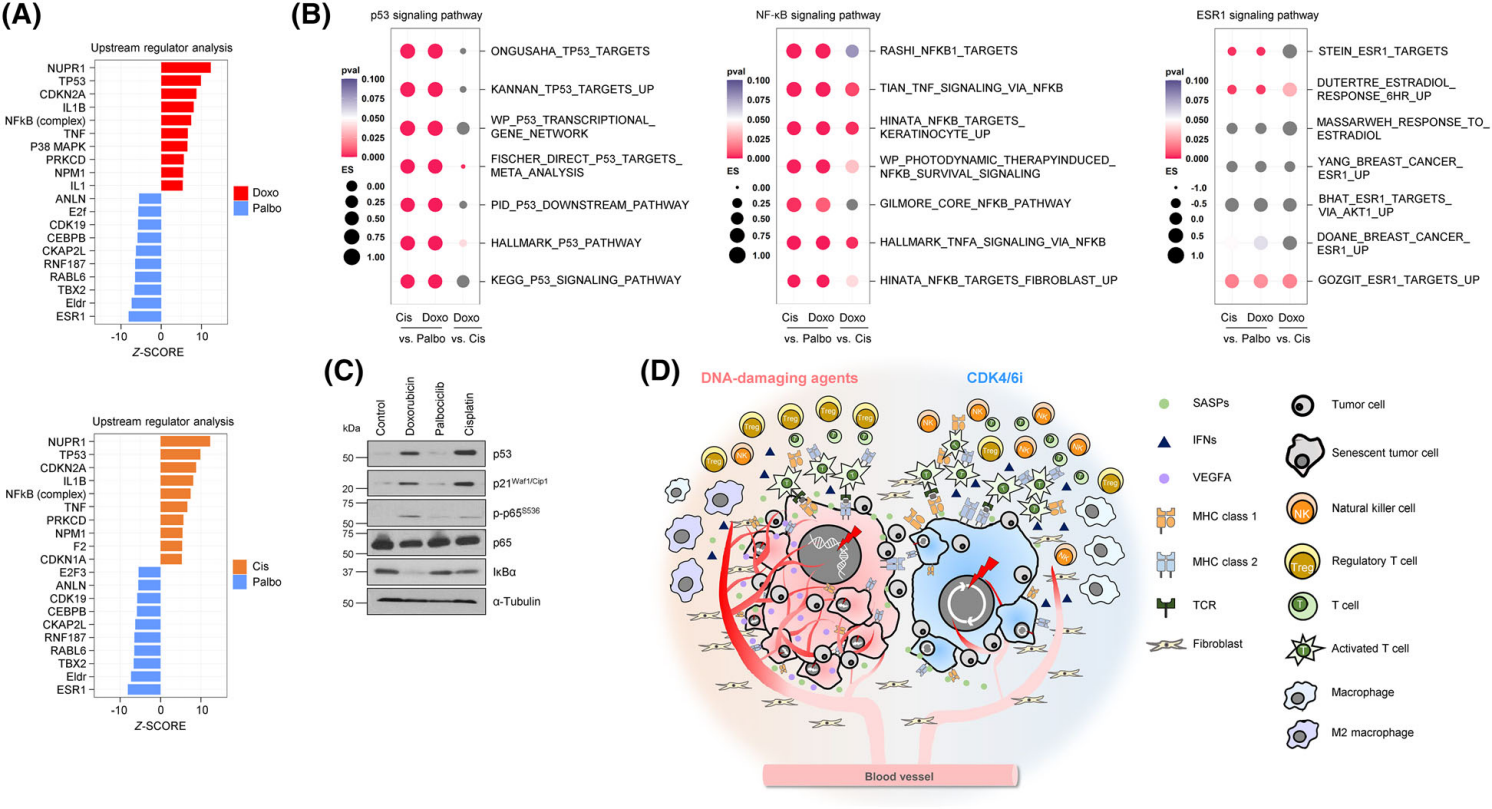
Exclusive activation of p53 and NF‐κB signaling in DNA‐damaging agent‐induced senescent cells. (A) IPA was performed to analyze upstream regulators and their expression levels were compared between TIS groups; Doxo vs. Palbo and Cis vs. Palbo. The top 10 regulators were ranked according to the *z*‐score with *P* ≤ 0.05. (B) A comparison of ES of p53, NF‐κB, and ESR1 signaling‐related gene sets among TIS groups with *P*‐value; Cis vs. Palbo, Doxo vs. Palbo, and Doxo vs. Cis. (C) MCF‐7 cells were treated with DNA‐damaging agents and CDK4/6i and harvested after 5 days. The p53, p21^Waf1/Cip1^ expression, and NF‐κB activation were determined by immunoblotting. α‐tubulin was used as a loading control. Data are representative of three (*N* = 3) independent experiments. (D) An illustration depicting the effect of DNA‐damaging agents and CDK4/6i‐induced senescence on the TME of breast cancer cells. Cis, Cisplatin; Doxo, Doxorubicin; Palbo, Palbociclib.

Since the transcriptome analysis demonstrated a definitive difference between DNA‐damaging agent‐ and CDK4/6i‐induced senescence, we wonder if the presence of distinct upstream regulators can be expected by IPA. *TP53* and *NF‐κB* complex were enriched in DNA‐damaging agents, while estrogen receptor (*ESR1*) was enriched in CDK4/6i (Fig. 6A). The presence of these expected upstream regulators was supported by dotplot of gene sets associated with *TP53*, *TNF/NF‐κB*, and *ESR1* (Fig. 6B, Fig. S5). The strong stabilization of p53, expression of p21^Waf1/Cip1^, phosphorylation of p65, and degradation of IκB, which were observed only in DNA‐damaging agent‐induced senescent cells, supported the results of IPA analysis in MCF‐7 and HCC1428 cell lines (Fig. 6C, Fig. S3I). Therefore, considering that DNA damage response can activate NF‐κB signaling [66, 67], somewhat exclusive p53 and NF‐κB activation in DNA‐damaging agent‐induced senescence could explain the robust expression of pro‐inflammatory and pro‐angiogenic proteins. These results suggest that p53 and NF‐κB are not essential transcription factors for the expression of anti‐tumor immune‐related proteins in TIS.

## 4 Discussion

In the present study, although the extent of senescence elicited by DNA‐damaging agents and CDK4/6i was similar, CDK4/6i‐induced senescent cancer cells showed significantly lower expression of well‐known pro‐inflammatory SASP such as *IL‐6*, *CXCL1*, *CXCL8*, and *TGF‐β*. In a previous study, Coppe et al. demonstrated that overexpression of p16^INK4A^ induces premature cellular senescence without expression of SASP [18]. However, unlike the results of Coppe et al., other studies showed that CDK4/6i‐induced senescent cells expressed various SASP. Guan et al. observed that CDK4/6i (Palbociclib)‐induced senescent MEFs exhibited a more robust pro‐inflammatory gene expression profile than fibroblasts triggered to enter senescence by exposure to UV light or MMC; in addition, there was no DNA damage and p53 activation [19]. By contrast, Wang et al. demonstrated that CDK4/6i‐induced senescence in BJ fibroblasts was dependent on p53 activity, with no increase of NF‐κB activity or NF‐κB‐driven SASP molecules such as *IL‐6*, *CXCL1*, or *CCL5* [68]. However, in contrast with Wang et al study [68], we found increased expression of γH2AX and p21^Waf1/Cip1^ proteins, and strong enrichment of gene sets related to p53 activation, in cells exposed to DNA‐damaging agents only. Therefore, our results clearly suggest that CDK4/6i elicit cancer cell senescence without robust DNA damage or activation of p53 signaling. Since several studies show that DNA damage signals can activate NF‐κB and vice versa [67, 69], the stronger expression of a pro‐inflammatory SASP can be explained, in part, by NF‐κB activation by DNA‐damaging agents.

Increased expression of VEGF is a common characteristic of senescent human and mouse fibroblasts [18]. A recent study showed that combined treatment of a MAPK inhibitor (Trametinib) and Palbociclib induced senescence and these senescent pancreatic ductal adenocarcinoma cells showed increased secretion of pro‐angiogenic factors (VEGF, PDGFA/B, and FGF2) and MMPs (MMP2/3/7/9/10) [70]. However, in the present study, CDK4/6i‐induced senescence showed a decrease in VEGF expression. In addition, expression of other pro‐angiogenic factors was significantly lower in senescent breast cancer cells induced by CDK4/6i, whereas DNA‐damaging agent‐induced senescence increased expression of pro‐tumorigenic factors, including VEGF. Therefore, TIS after chemotherapy with conventional genotoxic agents may augment the survival and progression of non‐senescent residual cancer cells by promoting angiogenesis. In addition, considering the role of VEGF in inhibiting anti‐tumor immunity, senescent cancer cells induced by DNA‐damaging agents can promote a more potent pro‐tumorigenic immune microenvironment than CDK4/6i. Nevertheless, while some studies suggest that pro‐inflammatory and pro‐angiogenic SASP can enhance immune surveillance by NK cells [71], and improve the delivery of chemotherapeutic drugs [70], respectively, more research is needed to determine which types of chemotherapeutic agents (DNA‐damaging agents vs. CDK4/6i) can induce cancer cell senescence with more robust anti‐tumor immunomodulating capabilities.

If all the features of CDK4/6i‐induced senescence are less potent than those of DNA‐damaging agents, the implications of this study may not be significant. The most interesting point of this study is that CDK4/6i‐induced senescence still maintains the expression of SASP and ligands associated with anti‐tumor immunity. To elicit robust anti‐tumor immune responses, proper recruitment and tumor recognition of cytotoxic T cells are important. During CDK4/6i‐induced TIS, expression of genes related to antigen processing and presentation by MHC‐I (*HLA*, *B2M*, *TAP1*, and *ERAP1*), and those related to chemokines involved in T‐cell chemotaxis (*CXCL9*, *10*, *11*), was similar or even higher in senescence induced by CDK4/6i than DNA damaging agents. The results of a recent study showed that induction of senescence in cancer cells by various agents (Doxorubicin, Palbociclib, and Nutlin‐3A) increased expression of classical MHC‐I family and genes related to antigen processing [57] are consistent with our results. In addition, to the best of our knowledge, this is the first report to show that processes related to antigen presentation and T‐cell chemokine expression are activated more strongly by senescence induced by CDK4/6i than by senescence induced by DNA‐damaging agents.

According to our findings, activation of the NF‐κB pathway, as indicated by the phosphorylation of NF‐κB and the degradation of IκBα, was more prominent in senescent cells induced by DNA‐damaging agents. Conversely, the activation of STAT3, as assessed by its phosphorylation status, was comparatively higher in senescent cells induced by CDK4/6i. The differential activation of these signaling pathways could explain the distinct expression patterns of SASP and ligands induced by DNA‐damaging agents and CDK4/6i. The concept suggested by previous studies showing that pro‐ and anti‐tumorigenic SASP can be regulated separately by different signal pathways [72] can be applied to the results of the present study, although further studies are needed to identify which signal pathway is the main regulator for anti‐tumorigenic immunity during TIS.

Recent studies have shown that senescent cancer cells are highly immunogenic, and that immunization with senescent cancer cells [19], or nanovesicles [73] secreted by these cells, elicits strong anti‐tumor immune surveillance. From this point of view, further research comparing the induction of cancer cell senescence by different agents, and identifying the most potent immunogenic senescence inducers, will facilitate the development of more powerful therapeutic cancer vaccines based on senescent cancer cells. Nonetheless, experiments with mouse models, and analysis of human cancer samples treated with the above agents, need to be performed to validate our observation that DNA‐damaging agents and CDK4/6i‐induced senescence could have the potential to regulate the TME in different ways. Moreover, CDK4/6i are typically administered in conjunction with anti‐hormonal therapy, such as AI, in clinical practice for breast cancer treatment. Therefore, our results do not exclude the possibility that the reduced estrogen levels following AI treatment could influence the process of TIS by CDK4/6i in the cancer tissue of patients. Additionally, it should be noted that the results of the current study are limited to HR‐positive and HER2‐negative breast cancer cells and may need to be extended to other types of tumors.

## 5 Conclusion

We show that the SASP composition is highly dependent on the type of senescence inducer. CDK4/6i‐induced senescent cancer cells clearly showed less prominent expression of SASP and ligands associated with inflammation and angiogenesis, while comparable or even higher expression of SASP and ligands associated with anti‐tumor immunity compared to DNA‐damaging agent‐induced senescent cancer cells. These differences can elicit more favorable TME for cancer immune surveillance, potentially leading to better treatment outcomes (Fig. 6D). Taken together, our study provides a framework for a better understanding of TME modulation by TIS and may offer valuable insights into designing more effective senescence inducers as a therapeutic strategy.

## Conflict of interest

The authors declare no conflict of interest.

## Author contributions

DHL and MI performed the experiments, analyzed the data, and wrote the manuscript; JHC, YJP YHK, and SM participated in experiments and data interpretation; TJP and YWC designed the study, involved in supervision, project administration, interpretation, editing, and funding acquisition. All authors approved the final version of the manuscript.

### Peer review

The peer review history for this article is available at https://www.webofscience.com/api/gateway/wos/peer‐review/10.1002/1878‐0261.13541.

## Supporting information


**Fig. S1.** Effects on cell viability and CDK4/6i‐induced senescence with AI treatment. (A) MCF‐7 cells were treated with AI (Anastrozole and Letrozole) in a dose‐dependent manner for 5 days. Representative images are shown with a scale bar of 100 μm. Cell viability was measured after 5 days of AI treatment. (B) SA‐β‐gal staining was performed on MCF‐7 cells treated with Palbociclib (2 μm) or a combination of Palbociclib and AI (Anastrozole and Letrozole; 2.5, 5, 10, 20, and 30 μm). Representative images are shown with a scale bar of 200 μm, and the percentage of stained cells was quantified and presented as a bar graph. The *P*‐value was calculated using one‐way ANOVA. ***P < 0.001.
**Fig. S2.** Comparison of gene set enrichment analysis (GSEA) between control and TIS for immune responses and angiogenesis. The comparison of enrichment score (ES), obtained from GSEA, between control and therapy‐induced senescence (TIS) with gene sets related to (A) pro‐tumorigenic immune response, (B) angiogenesis, and (C) anti‐tumorigenic immune response; cutoff *P* ≤ 0.05.
**Fig. S3.** Differential expression of pro‐inflammatory SASP, pro‐angiogenic factors, and anti‐tumor immune‐related genes between DNA‐damaging agent‐ and CDK4/6i‐induced senescence in HCC1428 breast cancer cells. (A) HCC1428 cells were treated with DNA‐damaging agents (Cisplatin 5 μm and Doxorubicin 250 nm) and CDK4/6i (Palbociclib 5 μm) for 5 days and then SA‐β gal staining was performed. Representative images of SA‐β gal‐positive cells are shown with a scale bar of 200 μm. The percentage of stained cells was quantified and presented as a bar graph. Data are mean ± SD of three (*N* = 3) independent experiments. The *P*‐value was calculated using one‐way ANOVA. ***P < 0.001. (B) mRNA expression level of cell cycle‐related genes in senescent HCC1428 cells was measured by RT‐qPCR. Data are mean ± SD of three (*N* = 3) independent experiments. The *P*‐value was calculated using one‐way ANOVA. *P < 0.05 and **P < 0.01, and ***P < 0.001. (C, D) The cells were harvested after 5 days of drug treatment and proteins were detected with immunoblotting. Data are representative of three (*N* = 3) independent experiments. (E–G) The cells were harvested after 5 days of drug treatment, and proteins and mRNA expression levels were measured by performing RT‐qPCR. Data are mean ± SD of three (*N* = 3) independent experiments. The *P*‐value was calculated using one‐way ANOVA. TIS vs. Control; *P < 0.05, **P < 0.01, ***P < 0.001. Cis vs. Doxo and Palbo vs. Doxo; ^#^ *P* < 0.05. (H) The cells (4 × 10^5^ cells/100 mm dish) were treated and harvested after 5 days. The concentrations (pg·mL^−1^) of IL‐6, CXCL8, VEGFA, CXCL10, and IFN‐λ were measured by ELISA in conditioned media collected from senescent and control cells. Data are mean ± SD of three (*N* = 3) independent experiments. The *P*‐value was calculated using one‐way ANOVA. *P < 0.05 (TIS vs. Control) and ^#^ *P* < 0.05 (Cis vs. Doxo, Palbo vs. Doxo). (I) The drug‐treated HCC1428 cells were harvested after 5 days and p53, p21^Waf1/Cip1^ expression, and NF‐κB activation were determined by immunoblotting. β‐actin was used as a loading control. Data are representative of three (*N* = 3) independent experiments.
**Fig. S4.** Differential expression of pro‐inflammatory SASP, pro‐angiogenic factors, and anti‐tumor immune‐related genes in senescence induced by DNA‐damaging agents (Etoposide and Carboplatin) compared to CDK4/6i (Abemaciclib). (A) MCF‐7 cells were treated with DNA‐damaging agents (Etoposide 1 μm for 5 days and Carboplatin 75 μm for 5 days) and SA‐β gal staining was performed. Representative images of SA‐β gal‐positive cells are shown with a scale bar of 200 μm. The percentage of stained cells was quantified and presented as a bar graph. Data are mean ± SD of three independent experiments. The *P*‐value was calculated using one‐way ANOVA. ***P < 0.001. (B) Protein expression was determined by immunoblotting. β‐actin was used as a loading control. (C–E) MCF‐7 cells were treated with DNA‐damaging agents and CDK4/6i (Abemaciclib 1 μm for 5 days). The cells were harvested and subjected to RT‐qPCR for mRNA expression levels. Data are mean ± SD of three independent experiments. The *P*‐value was calculated using one‐way ANOVA. *P < 0.05, **P < 0.01, and ***P < 0.001 (TIS vs. Control).
**Fig. S5.** Comparison of gene set enrichment analysis (GSEA) between control and TIS for p53, NF‐κB, and ESR1 signaling pathway. The comparison of enrichment score (ES), obtained from GSEA, between control and therapy‐induced senescence (TIS) with gene sets related to (A) p53 signaling pathway, (B) NF‐κB signaling pathway, and (C) ESR1 signaling pathway; cutoff *P* ≤ 0.05.

**Table S1.** List of Primer Sequences.

## Data Availability

The data analyzed in this study are included either in this article or in additional files. Datasets for RNA‐seq have been deposited in GEO under accession No. GSE229264. https://www.ncbi.nlm.nih.gov/geo/query/acc.cgi?acc=GSE229264.
