# Supplementary material for: CDK4/6 inhibitors induce breast cancer senescence with enhanced anti‐tumor immunogenic properties compared with DNA‐damaging agents
Source: Mol Oncol. 2023 Nov 2;18(1):216–32. doi: 10.1002/1878-0261.13541 (PMC10766199; doi:10.1002/1878-0261.13541)

Fig. S1. Effects on cell viability and CDK4/6i-induced senescence with AI treatment

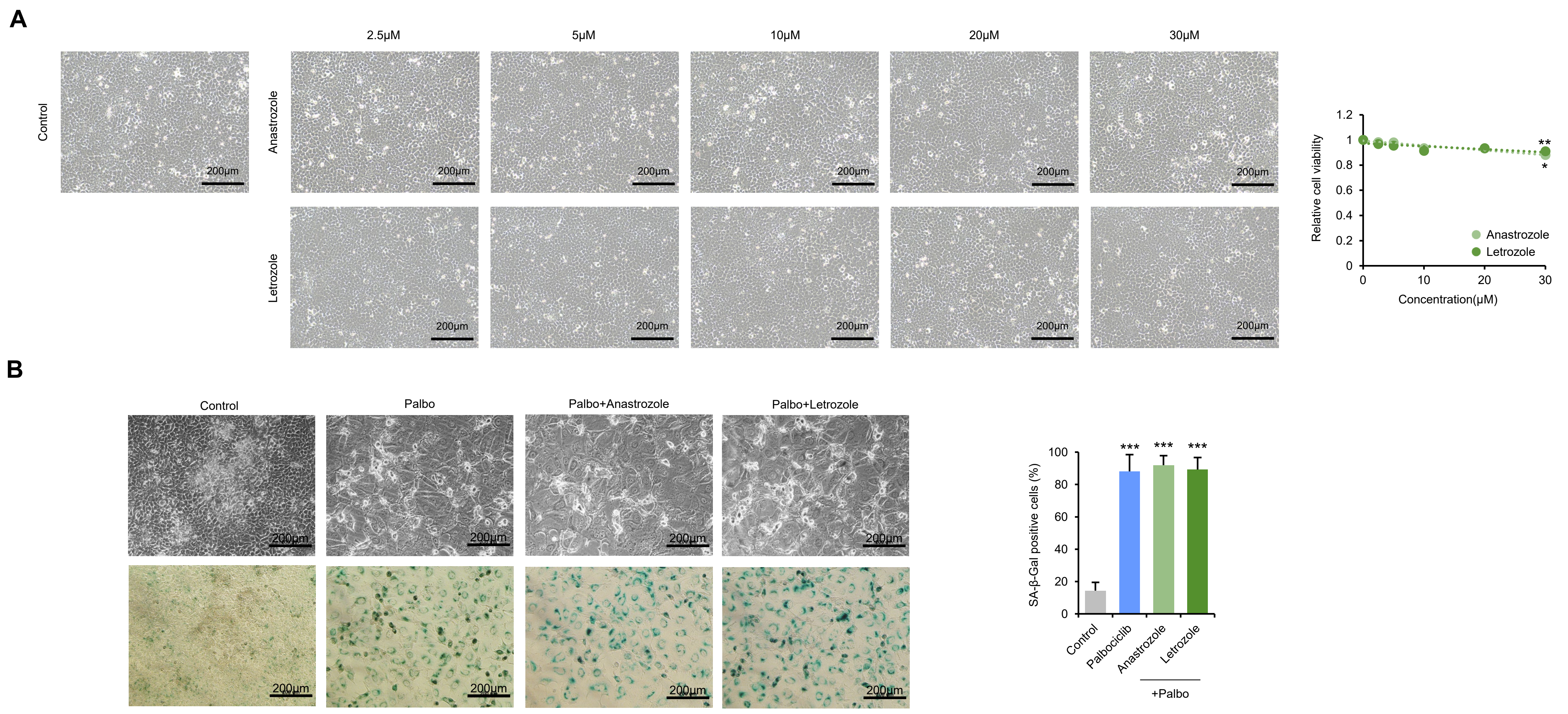

Fig. S2. Comparison of GSEA between control and TIS for immune responses and angiogenesis

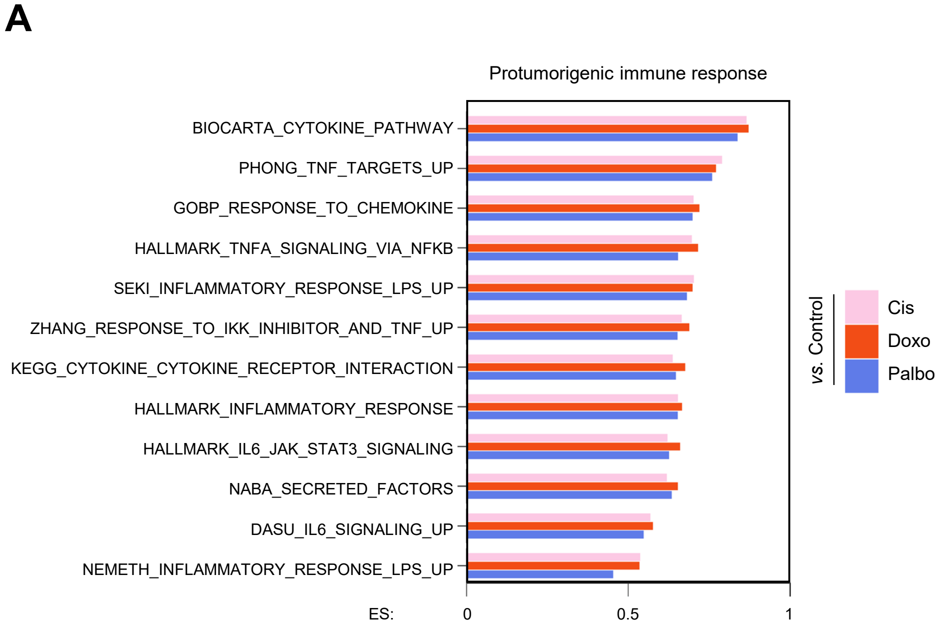

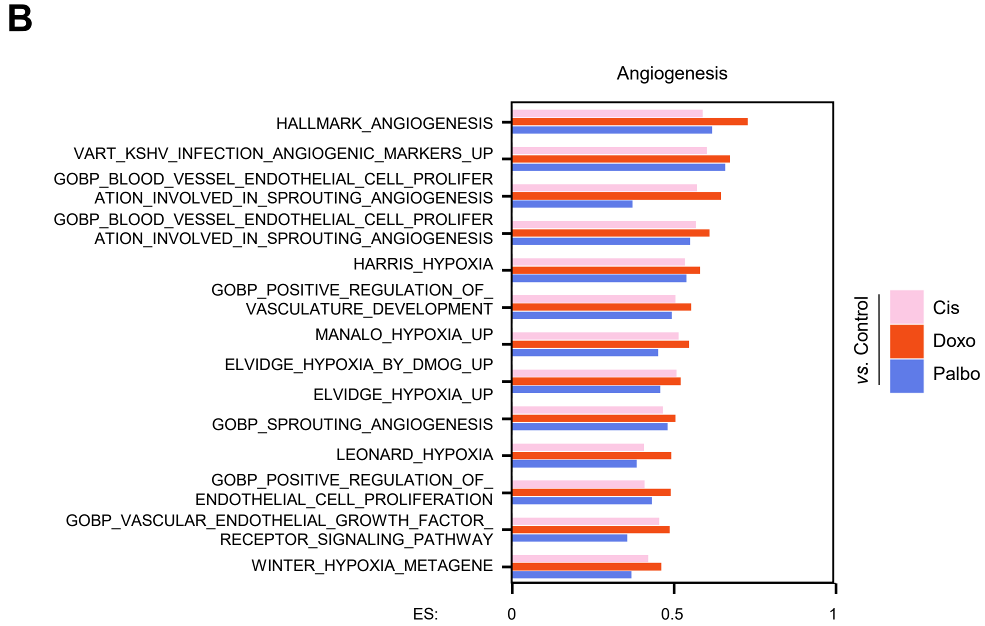

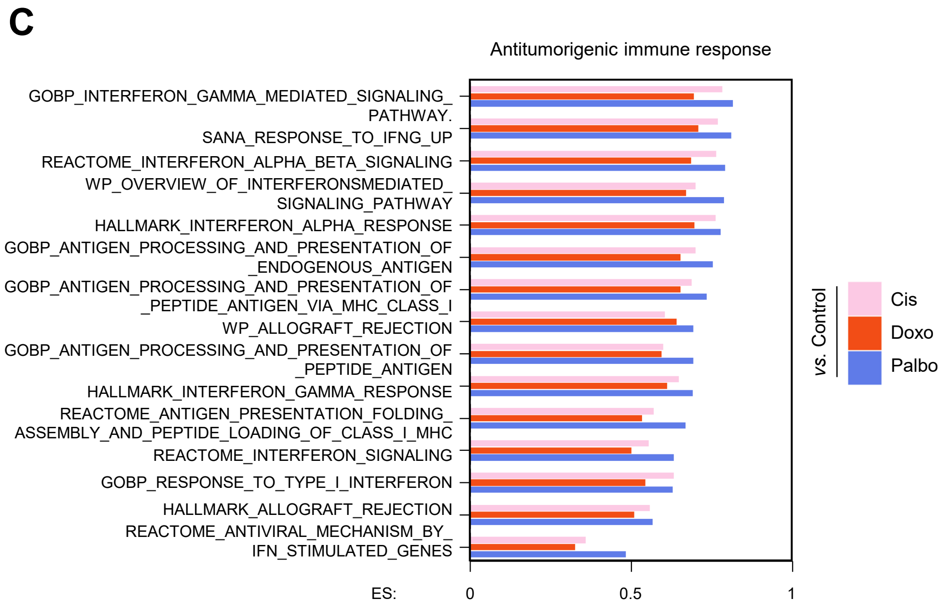

Fig. S3. Differential expression of pro-inflammatory SASP, pro-angiogenic factors, and anti-tumor immune-related genes between DNA-damaging agent- and CDK4/6i-induced senescence in HCC1428 breast cancer cells

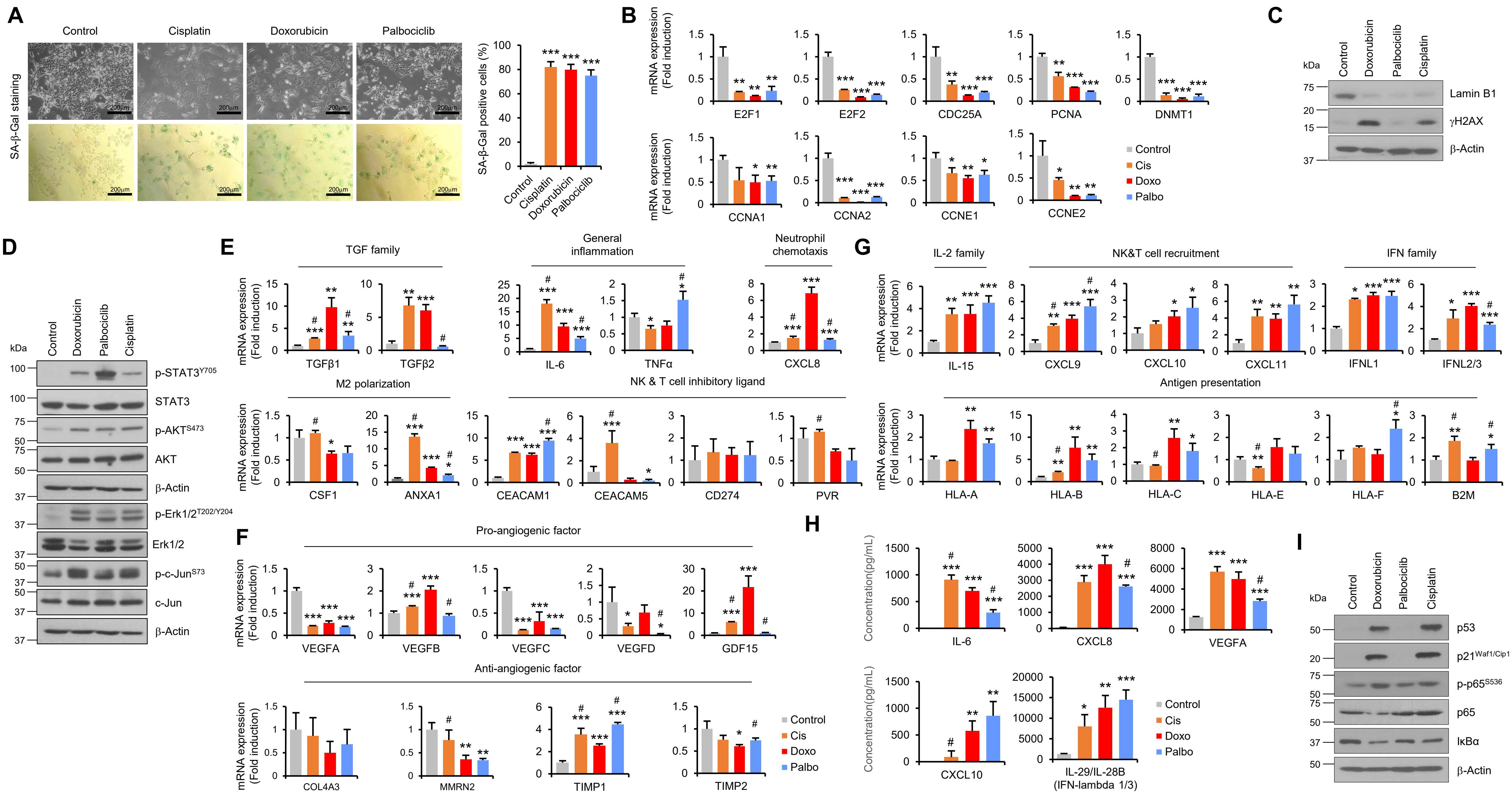

Fig. S4. Differential expression of pro-inflammatory SASP, pro-angiogenic factors, and anti-tumor immune-related genes in senescence induced by DNA-damaging agents (Etoposide and Carboplatin) compared to CDK4/6i (Abemaciclib)

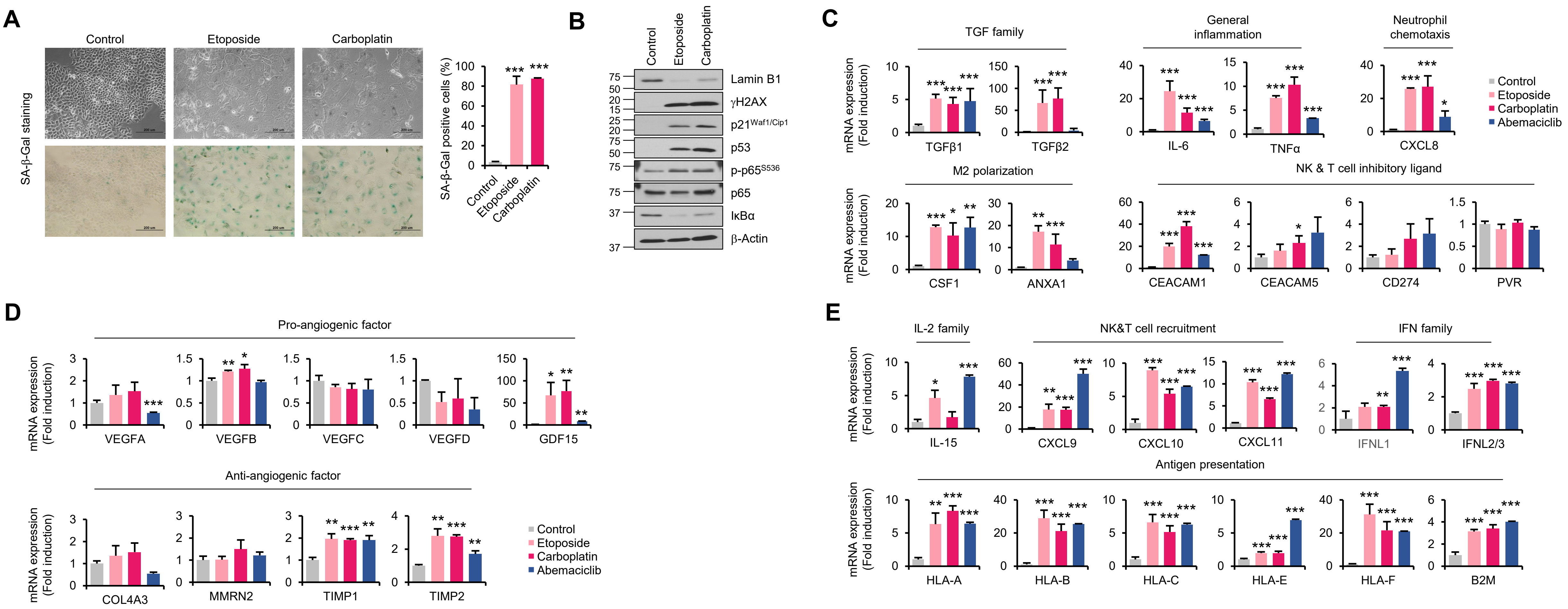

Fig. S5. Comparison of GSEA between control and TIS for p53, NF-κB, and ESR1 signaling pathway

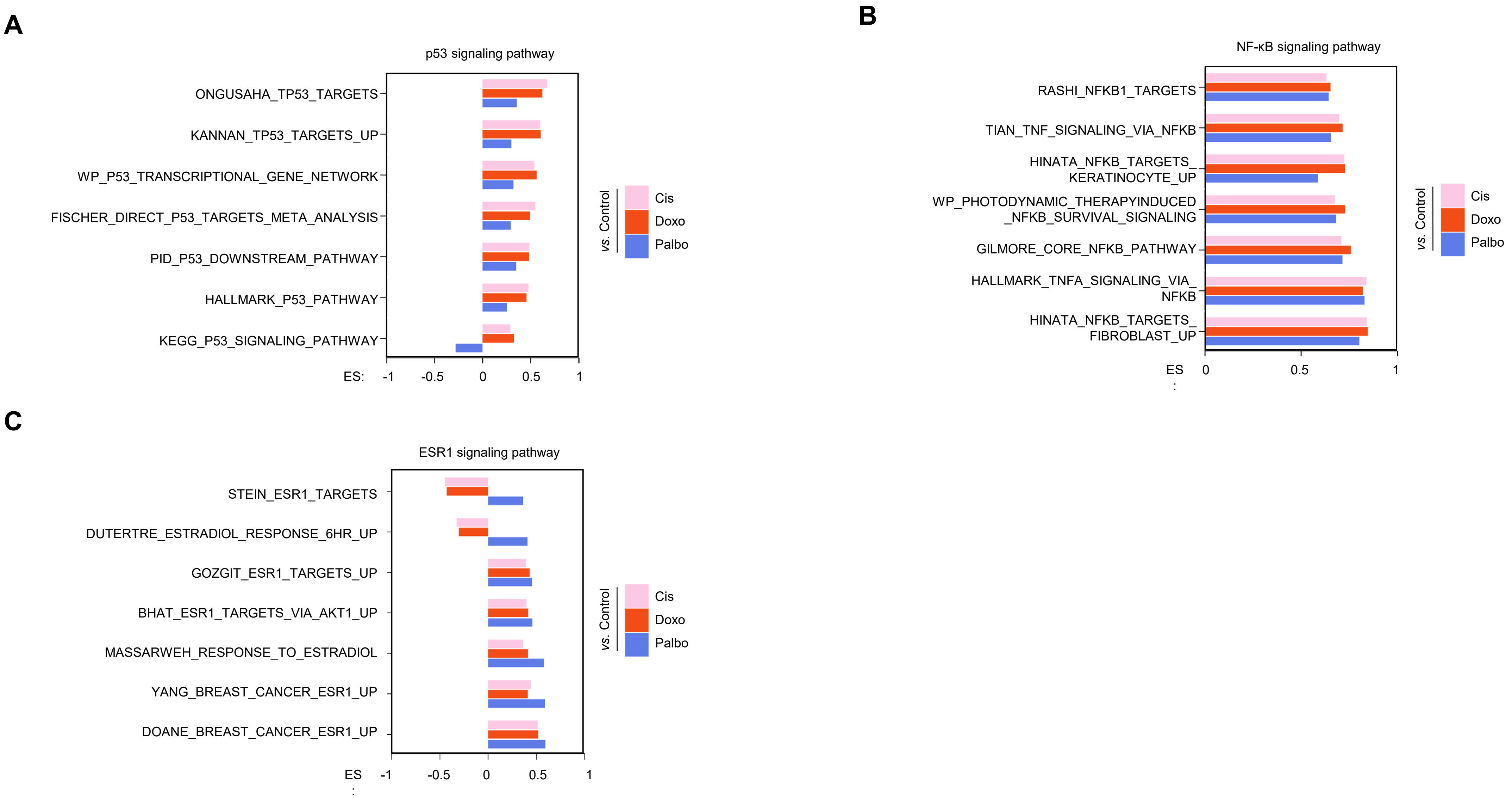

Supplement: Supplementary file 1 — Fig. S1. Effects on cell viability and CDK4/6i‐induced senescence with AI treatment. (A) MCF‐7 cells were treated with AI (Anastrozole and Letrozole) in a dose‐dependent manner for 5 days. Representative images are shown with a scale bar of 100 μm. Cell viability was measured after 5 days of AI treatment. (B) SA‐β‐gal staining was performed on MCF‐7 cells treated with Palbociclib (2 μm) or a combination of Palbociclib and AI (Anastrozole and Letrozole; 2.5, 5, 10, 20, and 30 μm). Representative images are shown with a scale bar of 200 μm, and the percentage of stained cells was quantified and presented as a bar graph. The P‐value was calculated using one‐way ANOVA. ***P < 0.001. Fig. S2. Comparison of gene set enrichment analysis (GSEA) between control and TIS for immune responses and angiogenesis. The comparison of enrichment score (ES), obtained from GSEA, between control and therapy‐induced senescence (TIS) with gene sets related to (A) pro‐tumorigenic immune response, (B) angiogenesis, and (C) anti‐tumorigenic immune response; cutoff P ≤ 0.05. Fig. S3. Differential expression of pro‐inflammatory SASP, pro‐angiogenic factors, and anti‐tumor immune‐related genes between DNA‐damaging agent‐ and CDK4/6i‐induced senescence in HCC1428 breast cancer cells. (A) HCC1428 cells were treated with DNA‐damaging agents (Cisplatin 5 μm and Doxorubicin 250 nm) and CDK4/6i (Palbociclib 5 μm) for 5 days and then SA‐β gal staining was performed. Representative images of SA‐β gal‐positive cells are shown with a scale bar of 200 μm. The percentage of stained cells was quantified and presented as a bar graph. Data are mean ± SD of three (N = 3) independent experiments. The P‐value was calculated using one‐way ANOVA. ***P < 0.001. (B) mRNA expression level of cell cycle‐related genes in senescent HCC1428 cells was measured by RT‐qPCR. Data are mean ± SD of three (N = 3) independent experiments. The P‐value was calculated using one‐way ANOVA. *P < 0.05 and **P < 0.01, and ***P < 0.001. [file MOL2-18-216-s001.pdf]
