# Supplementary material for: CDK4/6 inhibitors induce breast cancer senescence with enhanced anti‐tumor immunogenic properties compared with DNA‐damaging agents
Source: Mol Oncol. 2023 Nov 2;18(1):216–32. doi: 10.1002/1878-0261.13541 (PMC10766199; doi:10.1002/1878-0261.13541)
Supplement: Supplementary file 2 — Table S1. List of Primer Sequences. [file MOL2-18-216-s002.docx]

**Table S1: List of Primer Sequences**

| **Gene** | **Primers** |
| --- | --- |
| ANXA1 | Forward 5^′^-GATTCAGATGCCAGGGCCT-3^′^  Reverse 5^′^-GTGATTATTCCACATGTAATTGGTG-3^′^ |
| B2M | Forward 5^′^-TGCTGTCTCCATGTTTGATGTATCT-3^′^  Reverse 5^′^- TCTCTGCTCCCCACCTCTAAGT-3^′^ |
| CCL22 | Forward 5^′^-TAGGCTCTTCATTGGCTCAG-3^′^  Reverse 5^′^-ATTACGTCCGTTACCGTCTG-3^′^ |
| CCNA1 | Forward 5^′^-GCACACTCAAGTCAGACCTGCA-3^′^  Reverse 5^′^-ATCACATCTGTGCCAAGACTGGA-3^′^ |
| CCNA2 | Forward 5^′^-CTCTACACAGTCACGGGACAAAG-3^′^  Reverse 5^′^-CTGTGGTGCTTTGAGGTAGGTC-3^′^ |
| CCNE1 | Forward 5^′^-TGTGTCCTGGATGTTGACTGCC-3^′^  Reverse 5^′^-CTCTATGTCGCACCACTGATACC-3^′^ |
| CCNE2 | Forward 5^′^-CTTACGTCACTGATGGTGCTTGC-3^′^  Reverse 5^′^-CTTGGAGAAAGAGATTTAGCCAGG-3^′^ |
| CD274 | Forward 5^′^-GGCATCCAAGATACAAACTCAA-3^′^  Reverse 5^′^-CAGAAGTTCCAATGCTGGATTA-3^′^ |
| CDC25A | Forward 5^′^-TCTGGACAGCTCCTCTCGTCAT-3^′^  Reverse 5^′^-ACTTCCAGGTGGAGACTCCTCT-3^′^ |
| CEACAM1 | Forward 5^′^-CAGTCACCTTGAATGTCACCTATG-3^′^  Reverse 5^′^-GTTCCATTGATAAGCCAGGAGTAC-3^′^ |
| CEACAM5 | Forward 5^′^-AGGCCAATAACTCAGCCAGT-3^′^  Reverse 5^′^-GGGTTTGGAGTTGTTGCTGG-3^′^ |
| COL4A3 | Forward 5^′^-CCCGAGAGATACGCAGGTG-3^′^  Reverse 5^′^-AGTGCTGCCCAAATCTCCTCTG-3^′^ |
| CSF1 | Forward 5^′^-CCAGGAACAGTTGAAAGATCCA-3^′^  Reverse 5^′^-TTATCTCTGAAGCGCATGGTGT-3^′^ |
| CXCL1 | Forward 5^′^-AGCTTGCCTCAATCCTGCAT-3^′^  Reverse 5^′^-CCTCTGCAGCTGTGTCTCTC-3^′^ |
| CXCL11 | Forward 5^′^-TGGGATTTAGGCATCGTTGT-3^′^  Reverse 5^′^-CCTGGGGTAAAAGCAGTGAA-3^′^ |
| CXCL12 | Forward 5^′^-TGCCAGAGCCAACGTCAAG-3^′^  Reverse 5^′^-CAGCCGGGCTACAATCTGAA-3^′^ |
| CXCL2 | Forward 5^′^-TGCAGGGAATTCACCTCAAG-3^′^  Reverse 5^′^-TGAGACAAGCTTTCTGCCCA-3^′^ |
| CXCL8 | Forward 5^′^-ATGACTTCCAAGCTGGCCGTGGCT-3^′^  Reverse 5^′^-TCTCAGCCCTCTTCAAAAACTTCT-3^′^ |
| DNMT1 | Forward 5^′^-AGGTGGAGAGTTATGACGAGGC-3^′^  Reverse 5^′^-GGTAGAATGCCTGATGGTCTGC-3^′^ |
| E2F1 | Forward 5^′^-GGACCTGGAAACTGACCATCAG-3^′^  Reverse 5^′^- CAGTGAGGTCTCATAGCGTGAC-3^′^ |
| E2F2 | Forward 5^′^-CTCTCTGAGCTTCAAGCACCTG-3^′^  Reverse 5^′^-CTTGACGGCAATCACTGTCTGC-3^′^ |
| GDF15 | Forward 5^′^-CCGAAGACTCCAGATTCCGA-3^′^  Reverse 5^′^-CCCGAGAGATACGCAGGTG-3^′^ |
| HLA-A | Forward: 5′-ACCCTCGTCCTGCTACTCTC-3′  Reverse: 5′-CTGTCTCCTCGTCCCAATACT-3′ |
| HLA-B | Forward 5′-CAGTTCGTGAGGTTCGACAG-3′  Reverse 5′-CAGCCGTACATGCTCTGGA-3′ |
| HLA-C | Forward 5′-GGACAAGAGCAGAGATACACG-3′  Reverse 5′-CAAGGACAGCTAGGACAACC-3′ |
| HLA-E | Forward 5^′^-CGGCTACTACAATCAGAGCGAG-3^′^  Reverse 5^′^-AATCCTTGC CGTCGTAGGCGAA-3^′^ |
| HLA-F | Forward 5^′^-GGCAGAGGAATATGCAGAGGAGTT-3^′^  Reverse 5^′^-GGCAGAGGAATATGCAGAGGAGTT-3^′^ |
| IL11 | Forward 5^′^-GGACCACAACCTGGATTCCCTG-3^′^  Reverse 5^′^-AGTAGGTCCGCTCGCAGCCTT-3^′^ |
| IL15 | Forward 5^′^-ATGCTACTTTATATACGGAAA-3^′^  Reverse 5^′^-AGAAGACAAACTGTTGTTTGC-3^′^ |
| IL6 | Forward 5^′^-AAGCCAGAGCTGTGCAGATGAGTA-3^′^  Reverse 5^′^-TGTCCTGCAGCCACTGGTTC-3^′^ |
| MICB | Forward 5^′^-ACCTTGGCTATGAACGTCACA-3^′^  Reverse 5^′^-CCCTCTGAGACCTCGCTGCA-3^′^ |
| MMRN2 | Forward 5^′^-CAGAAAGACCTGGAAGCTCC-3^′^  Reverse 5^′^-GAAAAGCTGGCATAGAAGGC-3^′^ |
| PCNA | Forward 5^′^-CAAGTAATGTCGATAAAGAGGAGG-3^′^  Reverse 5^′^-GTGTCACCGTTGAAGAGAGTGG-3^′^ |
| PVR | Forward 5^′^-CCAACATGGAGGTGACGCAT-3^′^  Reverse 5^′^-GGCAGGTGTAGTTGCCTTCA-3^′^ |
| TGFβ1 | Forward 5^′^-TGCAGTTTTCCAGCAATGAG-3^′^  Reverse 5^′^-TGCGCTTGAGATCTTCAAA-3^′^ |
| TGFβ2 | Forward 5^′^-ACACTCAGCACAGCAGGGTCCT-3^′^  Reverse 5^′^-TTGGGACACGCAGCAAGGAGAAG-3^′^ |
| TIMP1 | Forward 5^′^-AGTGCTGCCCAAATCTCCTCTG-3^′^  Reverse 5^′^-TGCAGTTTTCCAGCAATGAG-3^′^ |
| TIMP2 | Forward 5^′^-ATGCACATCACC CTCTGTGA-3^′^  Reverse 5^′^-CTCTGTGACCCAGTCCATCC-3^′^ |
| VEGFA | Forward 5^′^-TTGCCT TGCTGCTCTACCTCCA-3^′^  Reverse 5^′^-GATGGCAGTAGCTGCGCTGATA-3^′^ |
| VEGFB | Forward 5^′^-TATATCCCAGTGGGGGAACA-3^′^  Reverse 5^′^-GACAAGGGATGGCAGAAGAG-3^′^ |
| VEGFC | Forward 5^′^-TGCCAGCAACACTACCACAG-3^′^  Reverse 5^′^-GTGATTATTCCACATGTAATTGGTG-3^′^ |
| VEGFD | Forward 5^′^-CTCGCAACGATCTTCGTCAAA-3^′^  Reverse 5^′^-CTGGAACAGAAGACCACTCTCATC-3^′^ |
| β-Actin | Forward 5^′^-CCCTGGCACCCAGCAC-3^′^  Reverse 5^′^-GCCGATCCACACGGAGTAC-3^′^ |
